# Controlled Oxidation of *n*‐Eicosane and Low‐Molecular‐Weight Polyethylene

**DOI:** 10.1002/cssc.71130

**Published:** 2026-10-12

**Authors:** Zhuoming Feng, Maura Gibbs, Anaise Thomas, Pedro Jimenez Antenucci, Hoda Shokrollahzadeh Behbahani, Karen I. Winey, Marisa C. Kozlowski, Karen I. Goldberg, John M. Vohs

**Affiliations:** ^1^ Department of Chemical and Biomolecular Engineering University of Pennsylvania Philadelphia Pennsylvania USA; ^2^ Department of Chemistry University of Pennsylvania Philadelphia Pennsylvania USA; ^3^ Department of Materials Science and Engineering University of Pennsylvania Philadelphia Pennsylvania USA

**Keywords:** acetic acid, autoxidation, bond cleavage, chemistry, ketone, oxygen, polyethylene, polymer chemistry

## Abstract

The aerobic oxidation of long‐chain hydrocarbons provides a direct route to introduce oxygen‐containing functionality into polyolefin backbones but is intrinsically coupled to C─C bond scission through radical autoxidation pathways. Here we examine the oxidation of *n*‐eicosane (C_20_) and low‐molecular‐weight polyethylene (PE4k) under O_2_ in the presence of 2,2′‐azobis(2‐methylpropionitrile) (AIBN) or acetic acid (HOAc). AIBN and HOAc efficiently initiate autoxidation at 90–125 °C, producing ketone and alcohol products at moderate conversion. Increasing temperature and oxygen partial pressure enhance overall conversion to oxidized products but promote C─C bond cleavage and formation of lower‐molecular‐weight products consistent with a β‐scission reaction pathway. Similar trends are observed for PE4k, where the additives promote CH_2_ group oxidation but reduce molecular weight at high temperatures or with high oxygen pressure. Low levels of oxygen incorporation were achieved with low chain scission at reduced oxygen partial pressure. The AIBN and HOAc reactions proceed via different radical initiations but are proposed to converge to a common autoxidation radical chain propagation mechanism. Across both substrates, oxygen concentration and reaction temperature control the balance between functionalization and polymer backbone cleavage, defining an operational window for selective oxidative modification of polyolefins.

## Graphical abstract

Controlled oxidation of *n*‐eicosane and polyethylene is achieved under mild conditions using AIBN and acetic acid as initiators. Oxygen functionalization selectively introduces ketone and alcohol groups, while limiting extensive carbon‐chain scission. The approach demonstrates tunable C─H functionalization of long‐chain hydrocarbons and provides a mild strategy for converting polyethylene into value‐added oxygenated materials.

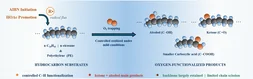

## 1 Introduction

Polyolefins are used in applications ranging from packaging to high‐strength fibers to advanced materials [1, 2, 3, 4]. They account for over 50% of global plastic production, with an annual output exceeding 200 million metric tons [5]. While there are many advantages of the use of polyolefins in these applications, it has also resulted in large waste streams of these materials. The chemical inertness of polyolefins, which is key to their utility, is also the major factor contributing to their persistence in the environment, making polymer waste accumulation a significant environmental concern [6, 7, 8]. In response, substantial efforts have been directed toward the reuse of this waste stream either through depolymerization or through chemical upcycling, which aims to transform the waste polyolefins into higher‐value products [9]. Existing approaches for the former include catalytic hydrogenolysis to produce short‐chain alkanes [10, 11, 12, 13, 14, 15, 16, 17] and photocatalytic depolymerization [18, 19, 20, 21, 22, 23], which can generate smaller molecules, such as fuels, waxes, or monomers. An alternative chemical upcycling strategy is the direct post‐functionalization of polyolefins to enhance their properties while preserving the polymer backbone. For example, incorporation of even low levels of oxygen‐containing functionalities have been shown to significantly alter polymer properties, improving adhesion, compatibilization, and processability [24, 25, 26]. Hartwig et al. reported that introducing only ~3.8 mol% oxygen‐containing functional groups into polyethylene increased the adhesion strength to aluminum by nearly 20‐fold compared with that of the pristine polymer [27]. Moreover, these oxygen‐containing functional groups were further utilized as reactive handles for subsequent chemical transformations, enabling the synthesis of a broad range of hydroxyl‐ and carbonyl‐functionalized polyethylene derivatives with expanded applications and enhanced material value [28]. Compared to traditional copolymerization, postsynthetic functionalization introduces new functional groups onto existing polymer backbones. This strategy mitigates the known risk in copolymerization syntheses of catalyst poisoning by polar functional groups of the monomer or the formation of products with a high degree of branching during the reaction [29, 30, 31].

Selective postpolymerization functionalization of polyolefins via stoichiometric or catalytic C─H functionalization, however, remains challenging. Current commercial approaches to polymer modification rely on harsh toxic conditions, stoichiometric oxidants, or transition‐metal catalysts, which both increase cost and can lead to undesired side reactions including chain scission, crosslinking, and loss of material properties. Developing mild, selective strategies that introduce functional groups while preserving polymer integrity therefore remains an important goal.

Aerobic oxidation represents an attractive approach because molecular oxygen is abundant, inexpensive, and atom economical. However, oxidation of saturated hydrocarbons proceeds through radical autoxidation pathways that are intrinsically coupled to backbone degradation. In polymer systems, this chemistry underlies thermal and photooxidative aging processes that lead to embrittlement and molecular weight reduction. Controlling the rate and mode of radical generation is therefore central to balancing functionalization and degradation. Studies of the aerobic oxidation of relatively small alkanes indicate, however, that such control is achievable. For example, Lloyd and coworkers have reported that thermal radical initiators such as 2,2′‐azobis(2‐methylpropionitrile) (AIBN) can promote the partial oxidation of *n*‐decane under mild conditions to produce ketones, alcohols, and carboxylic acids [32]. Extension of this approach to long‐chain hydrocarbons and polyethylene (PE) offers a potential route to introduce oxygen‐containing (carbonyl and hydroxyl groups) functionality into inert polymer backbones.

Carboxylic acids are also known to influence hydrocarbon oxidation in metal‐catalyzed systems, where they participate in radical generation and peroxide turnover [33, 34, 35, 36]. Under oxidative conditions, carboxylic acids may also engage in peroxide‐mediated pathways that promote autoxidation in the absence of added metal. Comparing traditional radical initiator‐driven and carboxylic acid‐promoted systems provides an opportunity to examine how the mode of radical generation can influence selectivity and polymer integrity. Model long‐chain alkanes such as *n*‐eicosane provide a useful framework for understanding these processes; they approximate the local chemical environment of PE while allowing for more facile product analysis due to their liquid state at room temperature. Translation of these insights to low‐molecular‐weight PE enables the assessment of functionalization and molecular weight distributions.

In this work, the aerobic oxidation of *n*‐eicosane (C_20_) and low‐molecular‐weight polyethylene (PE4k) under O_2_ using either AIBN or acetic acid (HOAc) is examined, with the goal of achieving 1%–5% functionalization of the carbons along the polymer chain. Reaction temperature, oxygen partial pressure, and promoter identity are systematically varied to quantify their effects on conversion, product distribution, and molecular weight. Ultimately, under relatively mild conditions, carbonyl and hydroxyl groups can be introduced into the polymer backbone in the presence of an initiator with controlled CH_2_ conversion. Although some degree of C─C bond cleavage is observed, crosslinking remains minimal in the PE systems studied. Notably, polymer chains retain substantial C─C backbone integrity while undergoing the introduction of carbonyl and hydroxyl functionalities at controlled levels of conversion. The balance between functionalization extent and chain integrity can be modulated by varying the reaction temperature or oxygen partial pressure, thus enabling use of the products in a wider range of applications [28].

## 2 Experimental Section

The hydrocarbon reactants used in this study were *n*‐eicosane (C_20_H_42_, 99%, Sigma–Aldrich), which will be referred to as C_20_, and polyethylene (*M*_w_ ~ 4000 Da and *M*_n_ ~ 1700 Da as reported by Sigma–Aldrich), which will be referred to as PE4k. Reactions were performed using 30 or 50 mL, temperature‐ and pressure‐controlled, stainless steel, magnetic stirred batch reactors (Figure S1a,b). For each run, *n*‐eicosane or PE4k was loaded directly into the reactor (or a borosilicate glass vial placed in the reactor) along with a specified amount of radical initiator (AIBN, ABCN, and HOAc). The reactions were pressurized with a desired pressure of oxygen, and heated. At the end of reaction, the reactor was quenched in an ice bath.

The solid products obtained for reaction of C_20_ were collected and dissolved in toluene, and then the composition was determined using a gas chromatography–mass spectrometry (GC–MS). The solid products produced when using PE4k as the reactant and some C_20_ solid products were collected and characterized using ^1^H nuclear magnetic resonance (NMR) spectroscopy to determine the type and position of the functional groups. Fourier‐transform transform infrared (FTIR) spectroscopy was also used to characterize reaction products. The average molecular weight and dispersity (*Ð*) of the neat and functionalized PE samples were determined by high‐temperature gel permeation chromatography with infrared detection (HT GPC‐IR, Polymer Char). Additional details of the experimental methods and procedures are provided in Supporting Information.

## 3 Results

### 3.1 AIBN‐Promoted Partial Oxidation of *n*‐Eicosane

As noted in the Section 1, Lloyd and coworkers demonstrated that *n*‐decane can be oxidized to produce a range of oxygenated products such as ketones, alcohols, and carboxylic acids under relatively mild conditions, 70 °C in air, using the radical initiator AIBN [32]. To examine whether this reaction can be extended to longer chain alkanes and used for controlled introduction of oxygen‐containing functional groups along the backbone of PE, the aerobic oxidation of C_20_ and a low‐molecular weight PE4k (*M*_w_ ~ 4000/*M*_n_ ~ 1700 Da) were investigated in the presence of AIBN. C_20_ was used as a model compound for PE as the products could be analyzed by GS–MS, thereby aiding in mapping out of the reaction pathways.

For a reaction of C_20_ (3 g, 0.01 mol) with AIBN (50 mg, 0.3 mmol) at 90 °C under 0.5 MPa O_2_ for 10 h in a batch reactor, 10.6% of the C_20_ was converted to an oxygenated product as determined by GC–MS (Table 1, entry 4). Figure S2 displays the GC–MS trace of the reaction products, and Figures S3 and S4 display the mass spectrum of the two primary products. These data allowed the reaction products to be identified as ketones (C_20_‐one) and alcohols (C_20_‐ol) and show that for these reaction conditions, only singly oxygenated products and trace amounts of chain scission products were produced.

**TABLE 1:** C_20_ products—conversion and selectivity at 90 and 110 °C with and without AIBN.

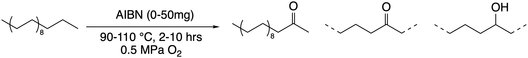								
Entry	Conditions	Initiator amount	C_20_ conversion% (based on GC–MS)^a^	CH_2_ conversion% (based on GC–MS)	CH_2_ conversion% (based on NMR)	Selectivity%		
						2‐one	Other‐one	Alcohol
1	90 °C, 0.5 MPa O_2_, 10 h	0 mg AIBN	0	0	0	0	0	0
2	90 °C, 0.5 MPa He, 10 h	50 mg AIBN	0	0	0	0	0	0
3	90 °C, 0.5 MPa O_2_, 2 h	50 mg AIBN	7.5	0.4	0.2	25	42	33
4	90 °C, 0.5 MPa O_2_, 10 h	50 mg AIBN	10.6	0.5	0.5	39	39	22
5	90 °C, 0.5 MPa O_2_, 10 h	100 mg AIBN	16.6	0.8	1.0	37	31	32
6	110 °C, 0.5 MPa O_2_, 5 h	0 mg AIBN	2.5	0.1	0.1	25	75	0
7	110 °C, 0.5 MPa O_2_, 5 h	50 mg AIBN	18.9	0.9	0.9	40	49	11

a Selected reactions were repeated and the experimental error for the C_20_ conversions based on GC–MS is estimated to be ±0.5%.

Two control experiments were also conducted. In the first, 3 g of C_20_ with added AIBN (50 mg) was heated in the batch reactor (90 °C, 10 h) in the presence of an inert gas (He, 0.5 MPa). Under these conditions, no reaction was observed, demonstrating the thermal stability of C_20_ at this temperature. The second control experiment was a repeat of the C_20_ oxidation run described above but without added AIBN initiator. The GC–MS trace of the reactor effluent for this run is displayed in Figure S5 and shows that only a trace amount (<0.1% conversion) of oxidation products was produced. The results obtained with and without AIBN demonstrate that AIBN is an effective initiator for the C_20_ oxidation reaction. Also note that the 10.6% C_20_ conversion is higher than the theoretical conversion expected (5.7%) for stoichiometric reaction with the radicals produced from 50 mg of AIBN (see calculation in Supporting Information).

Since homolysis of AIBN is known to commence at ~65 °C [37, 38], it may not be the most effective initiator for reaction at 90 °C. Furthermore, a higher reaction temperature will be required for oxidation of PE, which melts at temperatures greater than 100 °C. For this reason, the use of a more stable initiator, 1,1′‐azobis(cyclohexane carbonitrile) (ABCN), which undergoes homolysis at ~85 °C [39], was investigated. Using the same reaction conditions and molar amount of the initiator as in the AIBN run (3 g C_20_, 80 mg ABCN (0.3 mmol), 0.5 MPa O_2_ for 10 h), the C_20_ conversion to alcohol and ketone products using ABCN was 10.3%, which is essentially the same as that obtained for AIBN. Meanwhile, GC–MS analysis of the products showed that the composition was nearly the same as that obtained in the runs conducted with AIBN. For this reason, AIBN was used in the rest of the study.

The influence of the initiator concentration on the reaction rate was also assessed by increasing the initial amount of AIBN to 100 mg. As shown in entry 5 of Table 1, doubling of the AIBN concentration resulted in an increase in the C_20_ conversion to the alcohol and ketone products from 10.6% to 16.6%.

The product assignments for C_20_ oxidation runs using 50 and 100 mg of AIBN were further confirmed via FTIR and ^1^H‐NMR spectroscopic analyses. The C═O stretching region of the FTIR spectrum of the products obtained from the 100 mg run is displayed in Figure S6. Note that a peak at 1710 cm^−1^, characteristic of the C═O stretch of a ketone group, is present in the spectra of the oxidized sample. The ^1^H‐NMR spectrum of the reactor effluent following reaction of C_20_ with 100 mg of AIBN and 0.5 MPa O_2_ at 90 °C for 10 h is displayed in Figure S7. Peak assignments are also included in the figure. Notable features in the ^1^H‐NMR spectrum include resonances at 2.4 ppm (─CH_2_─C(O)─CH_2_─) and 3.6–4.0 ppm (─CH─OH─), which are attributed to hydrogens on carbons next to carbonyl and hydroxyl groups. Additionally, signals for the α‐ketone protons in the methyl group of methyl ketones (R─C(O) ─CH_3_) at 2.1–2.2 ppm allow ketone functionalities at the C_2_ position to be distinguished from those at other positions along the alkyl chain. Integration of ^1^H‐NMR spectral data also allow the total fraction of CH_2_ groups that have undergone reaction to be determined. Quantitative analysis of the GC–MS and ^1^H‐NMR data for this and several other runs with C_20_ is displayed in Table 1. As shown in entry 4 of Table 1, for reaction of C_20_ with 50 mg AIBN and 0.5 MPa O_2_ for 10 h at 90 °C, the selectivity to the ketone and alcohol products was 78% and 22%, respectively. It is also noteworthy that these data indicate that the C_2_ ketone product was favored over more interior positions along the chain.

Given that polyethylene processing requires temperatures above its melting point (>100 °C), the influence of elevated reaction temperature on the selectivity of C_20_ oxidation was investigated. The reaction was carried out at temperatures ranging from 90 to 110 °C and then 125 °C, with the O_2_ pressure remaining constant at 0.5 MPa. For reaction at 110 °C, a control experiment was performed in which AIBN was not added to the reactor. As shown by the GC trace of the products in Figure S8, for a reaction time of 5 h, 2.5% of the C_20_ underwent a reaction to produce oxygenated C_20_ products. ^1^H‐NMR spectral analysis of the products shows that this thermally induced oxidation reaction was selective for producing ketone functionalities (Table 1, entry 6). This result confirms reports that autoxidation of long‐chain hydrocarbons commences at moderately elevated temperatures, even without an initiator [40].

Adding 50 mg of AIBN radical initiator to the reaction mixture significantly increased the C_20_ conversion to 18.9% for a 5 h reaction time at 110 °C (Table 1, entry 7). The GC–MS data for this run (Figure S9) still showed selectivity for singly oxygenated products, although small peaks for doubly oxygenated products were also observed. The GC trace also shows that some lower molecular weight chain‐scission products were produced at this temperature. The product distribution for this run, determined by ^1^H‐NMR spectroscopy, is presented in Table 1 and shows that at 110 °C, the reaction was still selective for the production of ketone groups, which make up 89% of the product, with the rest being molecules containing OH groups. The overall CH_2_ group conversion was also close to 1%.

Increasing the reaction temperature to 125 °C had a much more dramatic effect on the overall conversion and the product distribution (Table 2). Since the reaction proceeds more rapidly at this higher temperature, we shortened the reaction time to only 1 h while maintaining the O_2_ pressure at 0.5 MPa.

**TABLE 2:** C_20_ products conversion and selectivity at 125 °C under different oxygen pressure.

Entry	Conditions	Initiators amount	NMR analysis	GC–MS analysis		
			CH_2_ conversion %	Overall conversion %	Conversion to C_20_‐ol and C_20_‐one products	Conversion to smaller oxygenate products %
1	125 °C, 0.5 MPa O_2_, 1 h	0 mg AIBN	0.2	7.4	5.2	2.2
2	125 °C, 0.5 MPa O_2_, 1 h	50 mg AIBN	5.4	62.8	29.6	33.2
3	125 °C, 0.1 MPa O_2_, 1 h	50 mg AIBN	1.7	39.2	25.1	14.1

For these conditions, in the absence of AIBN, the oxidation rate remained low, whereas upon the addition of 50 mg of AIBN, the reaction rate increased significantly, producing an overall C_20_ conversion of 62.8% (Table 2, entry 2). However, compared with reactions at 90 and 110 °C, significantly more chain scission products were formed in the reaction at 125 °C, which comprised 33.2% of the total reaction products (as determined by GC analysis, Figure S10). Analysis of the mass spectra of these small molecular weight products indicates that they are smaller carboxylic acids formed via C─C bond scission, which was also reported by Lloyd's work. To minimize chain scission, the oxygen pressure was reduced to 0.1 MPa while maintaining the reaction temperature at 125 °C and using 50 mg of AIBN. As shown in Table 2, for these reaction conditions, the C_20_ conversion decreased to 39.2% with chain scission products accounting for 14.1% of the total (entry 3).

### 3.2 Acetic‐Acid (HOAc)‐Promoted Partial Oxidation of *n*‐Eicosane

HOAc has frequently been reported to facilitate hydrocarbon oxidation in transition metal‐catalyzed systems [36, 41, 42, 43]. To determine whether it could promote oxidation under metal‐free conditions, the aerobic oxidation of *n*‐eicosane in the presence of HOAc without the addition of conventional radical initiators or added metal was also analyzed.

Although transition metals were not added to the acetic‐acid‐promoted oxidation reactions, the potential for acid etching of the metal of the reactor to produce metal ions in the reaction solution was a concern. As such, reactions were conducted in borosilicate glass vials placed within a stainless‐steel reactor, and only clean glassware and new (unused) stir bars were employed throughout. It was considered that carrying out the reaction in a glass vial potentially limits efficient heat transfer to the reactants. Indeed, a reaction of *n*‐eicosane and O_2_ (0.1 MPa) without added HOAc conducted directly in the metal reactor at 125 °C yielded ca. 20.1% oxidation products (Table 3, entry 7). When the same reaction was carried out in a glass vial within the reactor (Table 3, entry 2), no conversion was evident. These results are consistent with a mild improvement in heat transfer for reactions carried out directly in the reactor. However, to ensure that there were no acid interactions with the metal surface of the reactors that could either influence the results and/or damage the reactor, borosilicate vials were used for the HOAc‐initiated autoxidation reactions.

**TABLE 3:** C_20_ products conversion and selectivity at 110 and 125 °C with and without HOAc.

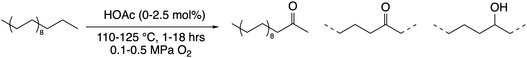							
Entry	Conditions	Acetic acid	C_20_ conversion% (based on GC–MS)	CH_2_ conversion% (based on NMR)	Selectivity%		
					2‐one	Other‐one	Alcohol
1	Unreacted C_20_	—	0	0	N/A	N/A	N/A
2	125 °C, 0.1 MPa O_2_, 18 h	—	0	0	N/A	N/A	N/A
3	110 °C, 0.1 MPa O_2_, 18 h	2.5 mol%	<1	<0.1	0	50	50
4	125 °C, 0.1 MPa O_2_, 18 h	0.5 mol%	4.1	0.1	33	25	42
5	125 °C, 0.1 MPa O_2_, 18 h	1.0 mol%	4.0	0.1	25	25	50
6	125 °C, 0.1 MPa O_2_, 18 h	2.5 mol%	4.5	0.1	33	11	56
7	125 °C, 0.1 MPa O_2_, 18 h^a^	—	20.1	0.4	57	31	12
8	125 °C, 0.5 MPa O_2_, 1 h	—	1.1	<0.1	81	19	0
9	125 °C, 0.1 MPa N_2_, 18 h	2.5 mol%	0	0	N/A	N/A	N/A

a Reaction run directly in stainless‐steel batch reactor.

When *n*‐eicosane (1.5 g, 0.005 mol) was heated to 125 °C in a borosilicate glass vial within a stainless‐steel reactor under a P_O2_ of 0.1 MPa with 2.5 mol% HOAc (rel. to O_2_), a 4.5% conversion to form ketone products was observed after 18 h (Table 3, entry 6). Figure S11 displays the GC–MS trace of the reaction products, showing negligible scission to smaller carbon chains. The ^1^H‐NMR spectrum (Figure S12) reveals conversion to 44% ketone and 56% alcohol products, indicating minimal selectivity for one over the other. A control reaction with no HOAc (Figure S13) or no O_2_ (Figure S14) yielded no conversion after heating for the same period, at the same temperature, and under the same pressure of O_2_ (Table 3). When heated at a lower temperature of 110 °C, negligible oxidation of *n*‐eicosane was observed even at similar concentrations of HOAc and O_2_ (Table 3, entry 3).

Optimization studies were conducted using the glass vials in the reactor, varying O_2_ pressure, temperature, and HOAc concentration. Reactions performed at 125 °C for 18 h in the presence of HOAc (0.5 mol%) reproducibly afforded ketone incorporation of approximately 4% relative to C_20_ molecules. Increasing HOAc concentration produced only marginal increases in conversion (4.5% with 2.5 mol% HOAc), with still minimal lower‐molecular‐weight products as seen by GC–MS. Increasing the oxygen partial pressure from 0.1 to 0.5 MPa resulted in higher conversion but led to an increase in lower‐molecular‐weight products as seen by GC–MS, suggesting that higher O_2_ loading may promote C─C bond cleavage. To better understand how the HOAc might be reacting with the substrate under the reaction conditions, reactions were tested in the presence of a radical inhibitor, butylated hydroxytoluene (BHT). A reaction of C_20_ at 125 °C for 18 h with both HOAc (2.5 mol%) and BHT (1 equiv. relative to HOAc) completely suppressed oxidative functionalization. This inhibition by BHT is consistent with a radical chain pathway for the autoxidation reaction.

Similar to the autoxidation results when the additive AIBN was used, GC–MS data for the products of the 2.5 mol% HOAc reactions revealed primarily singly oxygenated C_20_ species, with no significant formation of lower‐molecular‐weight fragments under optimized conditions. Product distributions and conversion were consistent with quantitative ^1^H‐NMR spectroscopic analysis. While the FTIR spectra did not show a pronounced OH stretch, resonances corresponding to alcohol functionalities were detected in the ^1^H‐NMR spectra.

### 3.3 Selective Oxidation of PE Using AIBN

Autoxidation studies with added AIBN were also conducted with PE4k samples at 90, 110, and 125 °C (Table 4). For these studies, the *M*_w_ and *M*_n_ of the PE4k batch that was used were determined to be 4000 and 1600 Da, respectively, by HT GPC‐IR (Table 5, entry 1). Due to the relatively low molecular weight of the PE4k sample, it has a melting temperature of ~90 °C, allowing for reactivity tests at this temperature. While this temperature is not expected to be practical for a higher molecular weight PE, the reactivity data at this temperature are useful in mapping out the trends in reactivity for PE.

**TABLE 4:** PE4k products conversion and selectivity at 90, 110, and 125 °C with and without AIBN.

Entry	Conditions	Initiators amount	CH_2_ conversion% (based on NMR)^a^,^b^	Selectivity%		
				2‐one	Other‐one	Alcohol
1	90 °C, 0.5 MPa O_2_, 10 h	0 mg AIBN	0.1	72	0	28
2	90 °C, 0.5 MPa O_2_, 10 h	50 mg AIBN	0.6	84	2	14
3	110 °C, 0.5 MPa O_2_, 10 h	0 mg AIBN	1.5	93	0	7
4	110 °C, 0.5 MPa O_2_, 10 h	50 mg AIBN	4.9	65	27	8
5	110 °C, 0.5 MPa O_2_, 10 h	100 mg AIBN	4.2	75	15	10
6	125 °C, 0.5 MPa O_2_, 4 h	0 mg AIBN	5.0	67	23	10
7	125 °C, 0.5 MPa O_2_, 4 h	50 mg AIBN	4.8	73	15	12

a Conversion is reported as the additional amount over the pre‐existing 1.2% oxidation in the PE4k substrate.

b Selected reactions were repeated and the experimental error based on 1H‐NMR measurements was ±0.2%.

**TABLE 5:** HT‐GPC data for PE4k reacted at 110 and 125 °C under 0.1 or 0.5 MPa O_2_ w/o AIBN.

Entry	Reaction conditions	*M*_w_, kDa	*M*_n_, kDa	*Đ* (*M*_w_/*M*_n_)	CH_3_/1000 C
1	Unreacted PE4k	4.0	1.6	2.5	37
2	110 °C, 0.5 MPa O_2_, 10 h	3.2	1.3	2.5	36
3	110 °C, 0.5 MPa O_2_, 10 h, 50 mg AIBN	1.9	0.7	2.5	42
4	125 °C, 0.5 MPa He, 4 h	4.0	1.6	2.5	38
5	125 °C, 0.1 MPa O_2_, 4 h	2.7	1.1	2.6	40
6	125 °C, 0.5 MPa O_2_, 4 h	2.3	0.9	2.5	42
7	125 °C, 0.5 MPa O_2_, 4 h, 50 mg AIBN	1.8	0.7	2.8	47

Table 4 summarizes the conversion and product distributions, as determined by ^1^H‐NMR spectroscopy, obtained for reaction of 3 g of PE4k under a variety of reaction conditions at temperatures between 90 and 125 °C (note that the as received PE4k contained 1.2% CH_2_ group oxidation and this value has been subtracted from the values in the table). These results are generally consistent with those observed for C_20_. In the absence of AIBN at 90 and 110 °C (entries 1 and 3 in Table 4), the oxidation rate of the PE4k was relatively low, whereas the addition of AIBN caused both the reaction rate and overall conversion of the CH_2_ groups to increase significantly (entries 2, 4, and 5 in Table 4). To further investigate these trends, the pressure in the batch reactor was monitored as a function of time. The data for reaction at 110 °C and an initial O_2_ pressure of 0.5 MPa with and without the AIBN initiator are presented in Figure 1. These data show that the O_2_ consumption rate increased significantly upon addition of the AIBN initiator.

**FIGURE 1:**
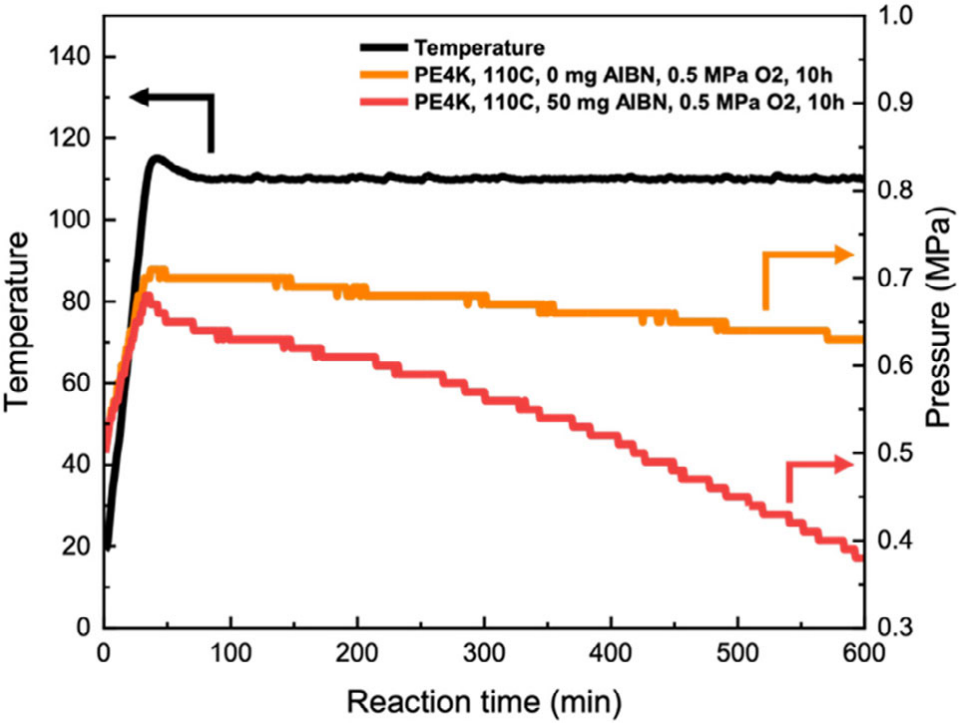
Pressure change profiles of O_2_ in the reactor at 110 °C and 0.5 MPa O_2_, with and without AIBN added in PE4k.

As was the case for C_20_, for reaction at 90 °C, the overall functionalization of the CH_2_ groups was quite small with conversions less than 1%. Note that even in the absence of AIBN, the reaction at 110 °C under 0.5 MPa O_2_ led to a higher extent of oxidation than at 90 °C with AIBN, reaching 1.5% CH_2_ conversion (Table 4, entry 3). Nevertheless, with the introduction of AIBN, the reaction proceeds at a faster rate, and a CH_2_ group conversion of 4.9% was obtained for reaction at 110 °C with 0.5 MPa of O_2_ and 50 mg of AIBN (Table 4, entry 4). In contrast to the behavior observed for the C_20_, for reaction of PE4k at 125 °C, the addition of AIBN did not enhance the reaction rate with the CH_2_ conversion remaining at ~5%. This indicates that the reaction can be thermally initiated at this temperature.

The reaction selectivity data still indicate a preference for reaction near the chain ends, but significant oxidation along the chain is also observed. Compared to free radical chain oxidations of hydrocarbons in the gas phase, the rates obtained here in the liquid phase are much lower, and as shown in Table 4, increasing AIBN amount did not lead to a proportional increase in the oxidation yield (entries 4 and 5 in Table 4). This relatively slow rate of the chain reaction in the liquid phase can be attributed to the cage effect. In molten polymers, the radicals generated from dissociation of the initiator will remain close to one another, increasing the propensity for their recombination prior to reacting with a polymer chain. The radicals can also be trapped through polymer entanglement rather than reacting with oxygen to propagate oxidation along the carbon backbone. These effects are well documented in previous studies of chain reactions in polymers [20, 44, 45, 46].

To determine whether the effects of AIBN, reaction temperature, and oxygen pressure on C─C bond scission are similar to those observed for C_20_, HT GPC‐IR was used to assess the extent of chain scission that occurred during oxidation reactions of PE4k at 110 and 125 °C. The individual HT GPC‐IR traces are presented in Figure 2a,b, and the number‐average molecular weight (*M*_n_) and dispersity (*Đ*) values obtained from these data are compiled in Table 5.

**FIGURE 2:**
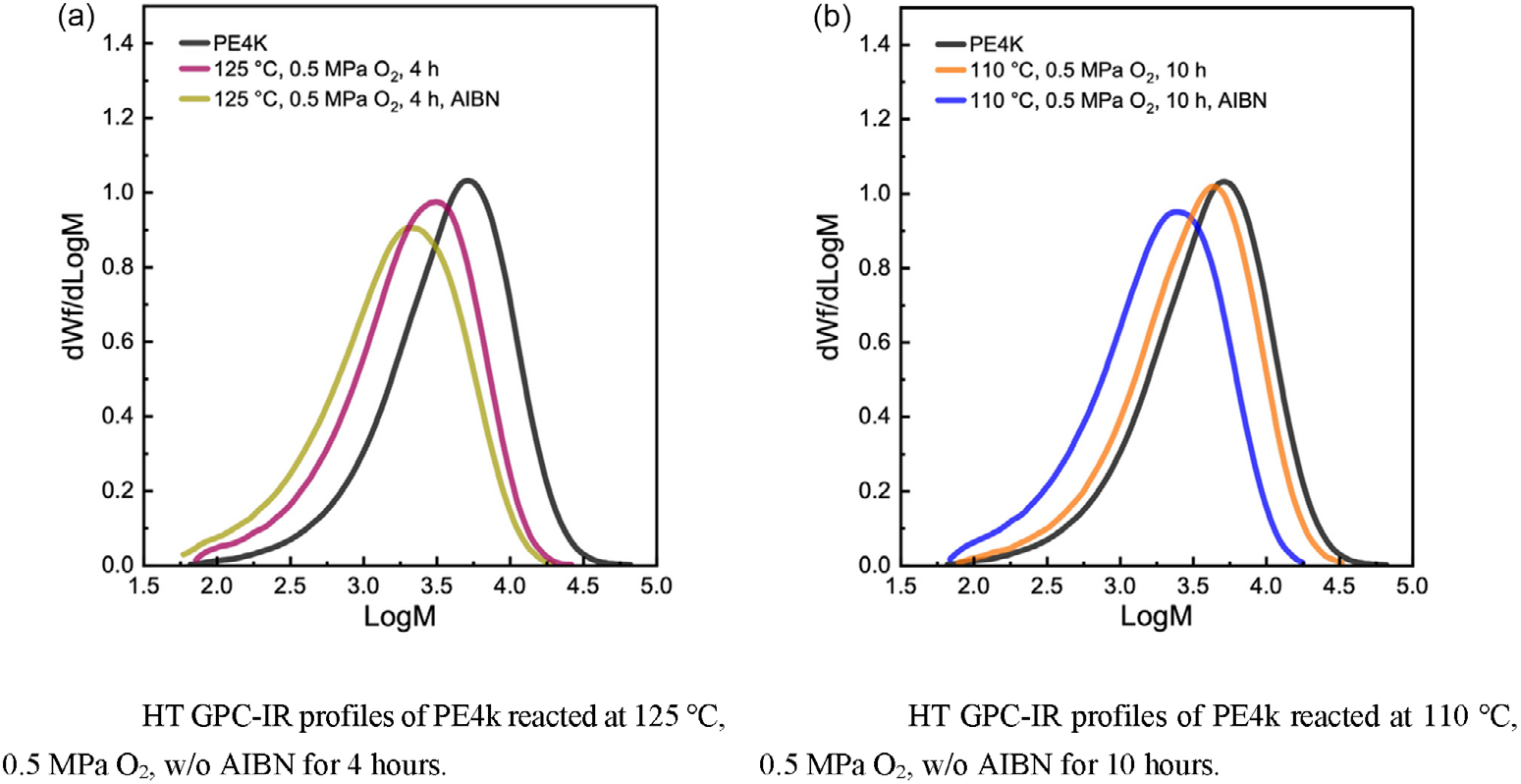
(a) HT GPC‐IR profiles of PE4k reacted at 125 °C, 0.5 MPa O_2_, with and without AIBN for 4 h. (b) HT GPC‐IR profiles of PE4k reacted at 110 °C, 0.5 MPa O_2_, with and without AIBN for 10 h.

Heating the PE4k to 125 °C for 4 h under 0.5 MPa He had no effect on the measured *M*_n_ and *Đ* thereby demonstrating the thermal stability of the polymer at this temperature (entries 1 and 4 in Table 5). In the presence of oxygen, however, the *M*_w_ decreased to varying extents depending on the reaction time and oxygen pressure (see *M*_w_ values in Table 5). In particular, when AIBN was included in the autoxidation reactions, the *M*_w_ dropped from 4.0 kDa to close to 1.8 kDa at both 110 and 125 °C (entries 3 and 7 in Table 5). The higher short chain branching (CH_3_/1000 C) value relative to unreacted PE4k further supports that AIBN promotes C─C bond scission.

Since it was observed in the C_20_ experiments that lower oxygen pressure reduced the extent of C─C bond cleavage (see above), the effect of lowering the O_2_ pressure from 0.5 to 0.1 MPa on the extent of polymer chain scission at 125 °C was also examined. AIBN was not used in these experiments since it was shown to be not effective in promoting the oxidation reaction at this temperature. Due to the lower O_2_ pressure, the majority of the O_2_ was consumed after only 4 h of reaction. To investigate the extent of chain scission at longer times, the batch reactor was recharged with O_2_ after 4 h, and the system was allowed to react for an additional 4 h.

The results of these experiments are shown in Table 6. As expected, lowering the oxygen pressure led to a noticeable decrease in CH_2_ conversion (from 5% to 3.4% at 0.5 and 0.1 MPa, respectively, after 4 h). However, upon repressurizing the reactor to restore the initial oxygen level, the CH_2_ conversion did not increase proportionally. Instead, a larger fraction of “other‐one” products was formed, which likely corresponds to species generated through C─C bond cleavage. This observation is consistent with the HT GPC‐IR results (Figure 3), which show that repressurization further decreased *M*_w_, suggesting additional chain scission events during the renewed high‐pressure oxidation period. Combining the *M*_w_ with the product conversion, it appears that the extent of C─C bond scission during the reaction follows the same trend as the CH_2_ conversion. In terms of optimization, to control the extent of C─C bond scission using AIBN as an initiator while achieving an acceptable conversion, 110 °C appears to be the optimal temperature.

**TABLE 6:** PE4k products conversion and selectivity at 125 °C without AIBN under different reaction time.

Entry	Conditions	CH_2_ conversion% (based on NMR)	Selectivity%		
			2‐one	Other‐one	Alcohol
1	125 °C, 0.1 MPa O_2_, 4 h	3.4	70	20	10
2	125 °C, 0.1 MPa O_2_, 4 h + 4h	4.4	64	28	8

**FIGURE 3:**
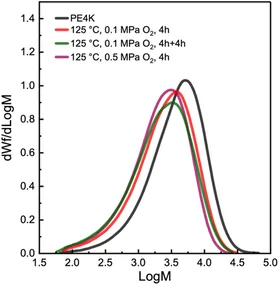
HT GPC‐IR profiles of PE4k reacted at 125 °C under 0.1 and 0.5 MPa O_2_ for 4 and 8 h.

### 3.4 HOAc Promoted Oxidation of PE4k

Experiments with HOAc as the additive to promote autoxidation of PE4k were also conducted. All reactions with HOAc were again performed at 125 °C in a glass vial within the reactor to minimize any potential reactivity of the acid with the stainless‐steel reactor. Several control reactions without acid were run directly in the reactor. For the HOAc reactions described below, the molecular weights of the batch of PE4k used in these experiments were determined to be *M*_w_ = 4000 and *M*_n_ = 1700 Da by HT GPC‐IR (Table 7, entry 1), and the initial percent of ketone per CH_2_ in the material was 0.8 mol% (Figure S15). For all CH_2_ conversions listed in Tables 8 and 9, the % conversion is reported as the additional amount over the pre‐existing 0.8% oxidation in the PE4k substrate.

**TABLE 7:** HT GPC‐IR data for PE4k reacted at 110 and 125 °C with and without HOAc.

Entry	Reaction conditions	*M*_w_, kDa	*M*_n_, kDa	*Đ* (*M*_w_/*M* *_n_*)	CH_3_/1000 C
1	Unreacted PE4k	4.0	1.7	2.5	36
2	110 °C, 0.1 MPa O_2_, 18 h, 2.5 mol% HOAc	3.9	1.7	2.3	37
3	125 °C, 0.1 MPa O_2_, 18 h	3.9	1.6	2.4	37
4	125 °C, 0.1 MPa O_2_, 18 h, 2.5 mol% HOAc	3.8	1.5	2.5	37
5	125 °C, 0.1 MPa O_2_, 18 h, 10 mol% HOAc	3.2	1.5	2.1	38
6	125 °C, 0.2 MPa O_2_, 18 h, 2.5 mol% HOAc	2.4	1.2	2.0	41
7	125 °C, 0.5 MPa O_2_, 18 h, 2.5 mol% HOAc	2.4	1.2	1.9	39
8	125 °C, 0.1 MPa O_2_, 18 h^a^	3.1	1.5	2.1	39

a Reaction run directly in stainless steel batch reactor.

**TABLE 8:** PE4k products conversion and selectivity at 110 and 125 °C with and without HOAc.

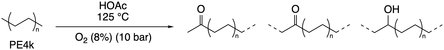							
Entry	Conditions	Acetic acid	CH_2_ conversion% (based on NMR)^a^^,^ b	Selectivity%			
				2‐one	Other‐one	Alcohol	
1	Unreacted PE4k	‐	0.8	54	46	0	
2	110 °C, 0.1 MPa O_2_, 18 h	2.5 mol%	<0.1	N/A	N/A	0	
3	125 °C, 0.1 MPa O_2_, 18 h	‐		61	39	0	
4	125 °C, 0.1 MPa O_2_, 18 h^c^	‐	1.2	51	44	5	
5	125 °C, 0.5 MPa O_2_, 4 h^c^	‐	1.1	79	11	10	
6	125 °C, 0.1 MPa O_2_, 18 h	2.5 mol%	0.7	71	21	8	
7	125 °C, 0.2 MPa O_2_, 18 h	2.5 mol%	1.3	41	51	8	
8	125 °C, 0.5 MPa O_2_, 18 h	2.5 mol%	2.3	41	49	10	
9	125 °C, 0.1 MPa O_2_, 18 h	10 mol%	0.9	37	56	7	
10	125 °C, 0.1 MPa N_2_, 18 h	2.5 mol%	0.1	51	49	0	

a Conversion is reported as the additional amount over the pre‐existing 0.8% oxidation in the PE4k substrate (Entry 1).

b Selected reactions were repeated and the experimental error based on ^1^H‐NMR measurements was ±0.1%.

c Reaction run directly in stainless‐steel batch reactor.

**TABLE 9:** Effects of acid identity on the oxidation of PE4k at 125 °C, 0.1 MPa O_2_, 18 h.

Entry	Acid	CH_2_ conversion% (based on NMR)^a^	Selectivity%		
			2‐one	Other‐one	Alcohol
1^b^	Acetic acid (2.5 mol%)	0.7%	71	21	8
2	Pivalic acid (2.5 mol%)	0.6%	31	54	15
3	*p*‐Toluene sulfonic acid (2.5 mol%)	<0.1%	N/A	N/A	N/A
4	Pyradinium[OTf] (1 mol%)	0.0%	N/A	N/A	N/A
5	Acetaldehyde (2.5 mol%)	0.6%	64	33	3
6	Sodium acetate (2 mol%)	0.4%	38	52	10
7	Trifluoroacetic acid (2.5 mol%)	0.4%	60	34	6

a Conversion is reported as the additional amount over the pre‐existing 0.8% oxidation in the PE4k substrate.

b Experiment was run in triplicate and values in parenthesis are calculated standard deviations.

Control experiments without O_2_ were initially conducted. Under 0.1 MPa of N_2_ and 2.5 mol% HOAc, no reaction was observed, as shown in ^1^H‐NMR spectrum (Figure S16). As shown in Table 8, autoxidation of the PE4k did proceed in the presence of oxygen at 125 °C. Note that compared with reaction in the glass‐vial, reaction of PE4k directly in the stainless‐steel reactor at 125 °C under 0.1 MPa O_2_ resulted in a higher conversion (Table 8, entries 3 and 4) and a more pronounced decrease in molecular weight (Table 7, entries 3 and 8). The lower extent of reaction observed in the glass vial is likely due to the actual reaction temperature for this setup being slightly lower than the nominal setpoint. When PE4k was subjected to aerobic oxidation in the presence of 2.5 mol% HOAc and 0.1 MPa O_2_, up to 0.7 mol% ketone was achieved after 18 h, as determined by ^1^H‐NMR analysis (Table 8, entry 6 and Figure S17), with the *M*_w_ decreasing by only 0.1 kDa (Table 7, entries 3 and 4). Increasing the concentration of HOAc from 2.5 mol% to 10 mol% resulted in only a small difference in the measured conversion (from 0.7% to 0.9% at 10 mol% HOAc, Table 8, entry 9, and Figure S18), which is almost within the estimated experimental error, but this modest increase was accompanied by a pronounced decrease in the *M*_w_ (Table 7, entry 5). These results were similar to those observed for the reaction of C_20_. Similar to the trend observed in the AIBN‐initiated experiments, increasing the O_2_ pressure led to higher conversion, accompanied by a more pronounced decrease in *M*_w_. The most substantial decrease occurred when the O_2_ pressure was increased from 0.1 to 0.2 MPa, with *M*_w_ decreasing from 3.8 to 2.4 kDa (Table 7, entries 4 and 6). In contrast, a further increase in O_2_ pressure from 0.2 to 0.5 MPa resulted in little additional decrease in *M*_w_ (Table 7, entry 7).

The effect of the identity of the acid was also investigated. Replacement of HOAc with pivalic acid ((CH_3_)_3_CCO_2_H) resulted in comparable levels of oxidation of the CH_2_ groups, 0.7 and 0.6 mol%, respectively. Sodium acetate (2 mol%) and trifluoroacetic acid (CF_3_CO_2_H, 2.5 mol%) likewise promoted a similar amount of oxidative functionalization. In contrast, use of a strong acid, *p*‐toluene sulfonic acid (CH_3_C_6_H_4_SO_3_H), which lacked carboxylate functionality, resulted in no detectable oxidative functionalization. Furthermore, with *p*‐toluene sulfonic acid, an increased *M*_w_ of 5800 Da, as determined by HT GPC‐IR, was observed consistent with acid‐promoted chain crosslinking leading to a larger hydrodynamic volume. Overall, these results with carboxylates and carboxylic acids indicate that it is the carboxylate moiety and not the proton of the acid that is critical to initiation of autoxidation. Acetaldehyde has been reported as a radical additive in base‐metal‐catalyzed chemistry, and under our conditions, this additive also promotes oxidation up to 0.6% conversion of the CH_2_ groups (Table 9, entry 5) [47].

Sequential repressurization with O_2_ (P_O2_ = 0.1 MPa) increased the overall ketone incorporation up to 1.7 mol%. However, the final repressurization step resulted in molecular weight decrease as measured by HT GPC‐IR, indicating C─C bond scission under prolonged high oxygen conditions (Figure 4).

**FIGURE 4:**
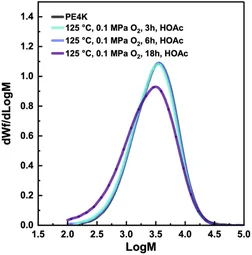
HT‐GPC profiles of PE4k reacted at 125 °C under 0.1 MPa O_2_ with HOAc and repressurized.

## 4 Discussion

The oxidation of *n*‐eicosane and polyethylene with molecular oxygen in the presence of either AIBN or HOAc results in functionalization of these substrates with ketone and alcohol groups. The results with both additives are consistent with radical chain autoxidation pathways. In both systems, carbon‐centered radicals are generated on the hydrocarbon backbone, followed by addition of O_2_ to form peroxyl radicals and subsequent hydrogen abstraction steps that propagate the oxidation.

### 4.1 Effect of Initiator Identity

In the case of AIBN, the results obtained here for partial oxidation of C_20_ are consistent with the aforementioned study of the oxidation of *n*‐decane by Lloyd and coworkers [32]. A possible radical chain mechanism for the reaction, similar to that proposed by Lloyd et al., is shown in Figure 5. The chain reaction is initiated by hydrogen abstraction from alkanes or the polymer backbone (─CH_2_─) by peroxy intermediates generated from the reaction of initiator‐derived radicals with molecular oxygen. In the chain propagation step, the generated alkyl radical reacts with O_2_ to produce alkyl peroxide radicals that abstract a hydrogen from an alkane reactant to produce another alkyl radical and an alkyl‐hydroperoxide species. The O─O bond in the alkyl‐hydroperoxide is relatively weak (~188 kJ/mol) compared to C─O (~376 kJ/mol) and O─H (~370 kJ/mol) bonds [48, 49] and this species undergoes decomposition to produce both alcohols and ketones, although the specific mechanistic steps to produce these functional groups are less clear.

**FIGURE 5:**
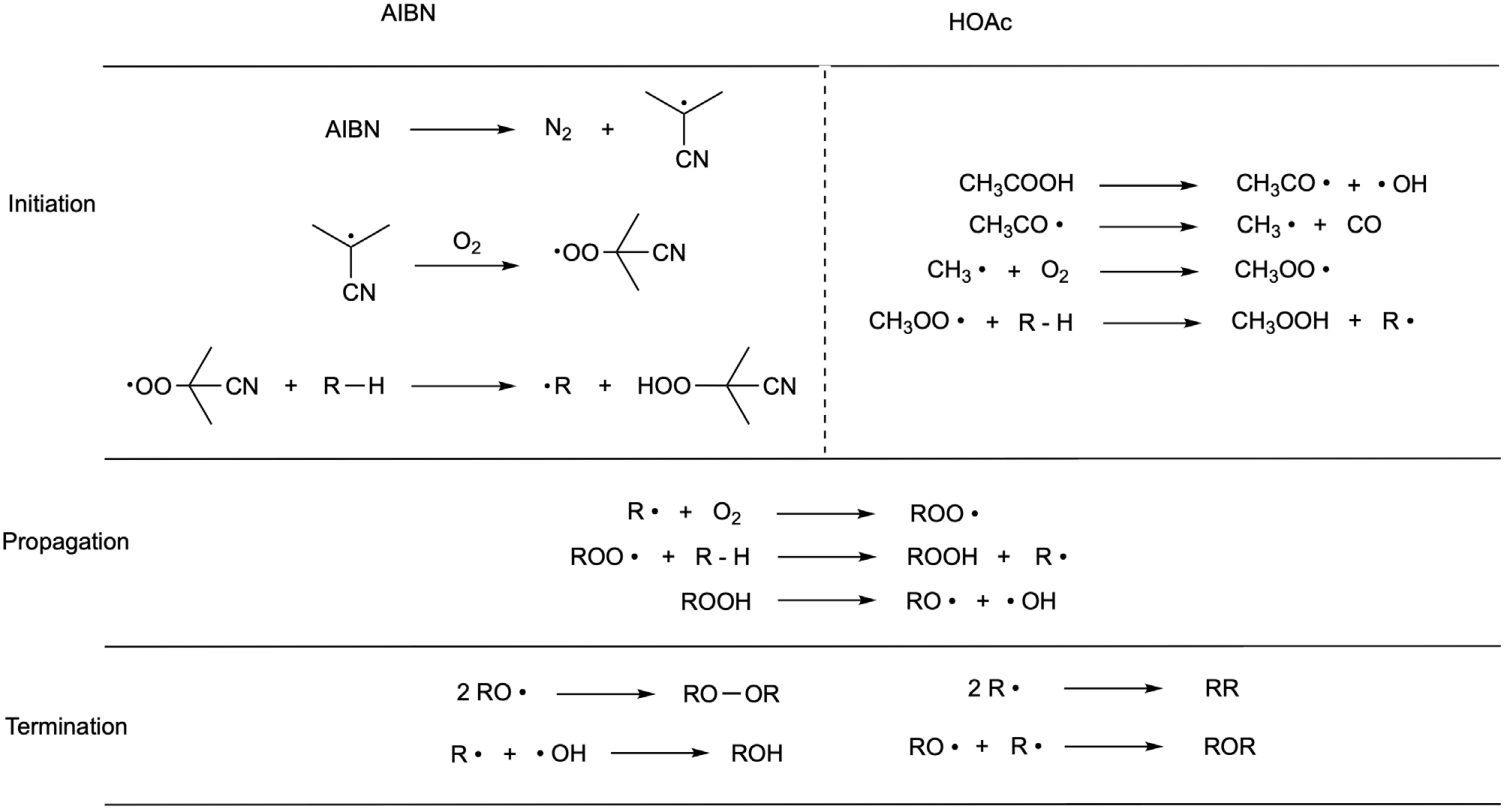
Proposed reaction scheme for the oxidation of hydrocarbons with AIBN and HOAc.

While the mechanism for radical autoxidation initiation by AIBN is well established, the HOAc initiation pathway is less precedented. Under elevated temperature and oxidative conditions employed here, HOAc is presumed to undergo homolytic processes that generate methyl (CH_3_•) radical species [35, 36], which are rapidly trapped by O_2_ to form methyl peroxyl radicals. The peroxyl radicals abstract a hydrogen from C─H bonds of the hydrocarbon substrates to generate carbon‐centered radicals (R•), thereby entering the same autoxidation cycle described above. In parallel, oxygen‐centered radicals from peroxide decomposition may contribute to additional hydrogen abstraction pathways.

Although reactions with AIBN and HOAc were conducted in different reactor configurations, the qualitative trends observed for both *n*‐eicosane and PE4k are consistent with an autoxidation framework in which temperature and O_2_ partial pressure contribute to the rate of oxidation. They both proceed via radical chain autoxidation mechanisms, with the most significant difference between them being the radical initiation steps.

### 4.2 Substrate Effects: Translation From Model Alkane to Polymer

The trends observed for *n*‐eicosane translated directly to PE4k, supporting the use of long‐chain alkanes as mechanistic surrogates for polyethylene oxidation. In both systems, the additives, AIBN and HOAc, increased the rate of oxygen incorporation. Higher temperature and oxygen pressure also increased overall conversion, but the increased conversion under these conditions correlated with increased C─C bond scission.

Notably, PE4k displayed greater sensitivity to oxidative conditions, likely due to pre‐existing oxidation and branching. As reported by Kumar and coworkers, preoxidized functional groups can lower adjacent C─H bond dissociation energies and accelerate autoxidation, leading to further oxygen insertion and fragmentation [50]. Thus, even in the absence of added initiator, PE4k undergoes measurable oxidation at 110–125 °C under oxygen, consistent with thermally induced autoxidation initiated at pre‐existing defects. This observation is particularly relevant for postconsumer materials, where intrinsic oxidation may facilitate further functionalization.

### 4.3 Trade‐Off Between Conversion and Backbone Integrity

Analysis of the C_20_ products for reactions at 110–125 °C by GC–MS and ^1^H‐NMR, spectral analysis revealed shorter chain alkanes and carboxylic acids (Figures S9, S10). HT GPC‐IR analysis of the reacted PE4k products also confirms that C─C bond cleavage occurred during the reaction, while the ^1^H‐NMR results further demonstrate the formation of carbonyl and hydroxyl groups. This trend was also observed in previous studies [51, 52]. Jalan et al. proposed that the carboxylic acid products are produced by a Korcek reaction pathway, resulting in chain scission like the one shown in Figure 6 [53]. These results indicate that it will be challenging to minimize chain scission while increasing oxygen functionality along the backbone under aerobic conditions. Fine tuning of the oxygen pressure and operation at temperatures below 125 °C will be critical.

**FIGURE 6:**
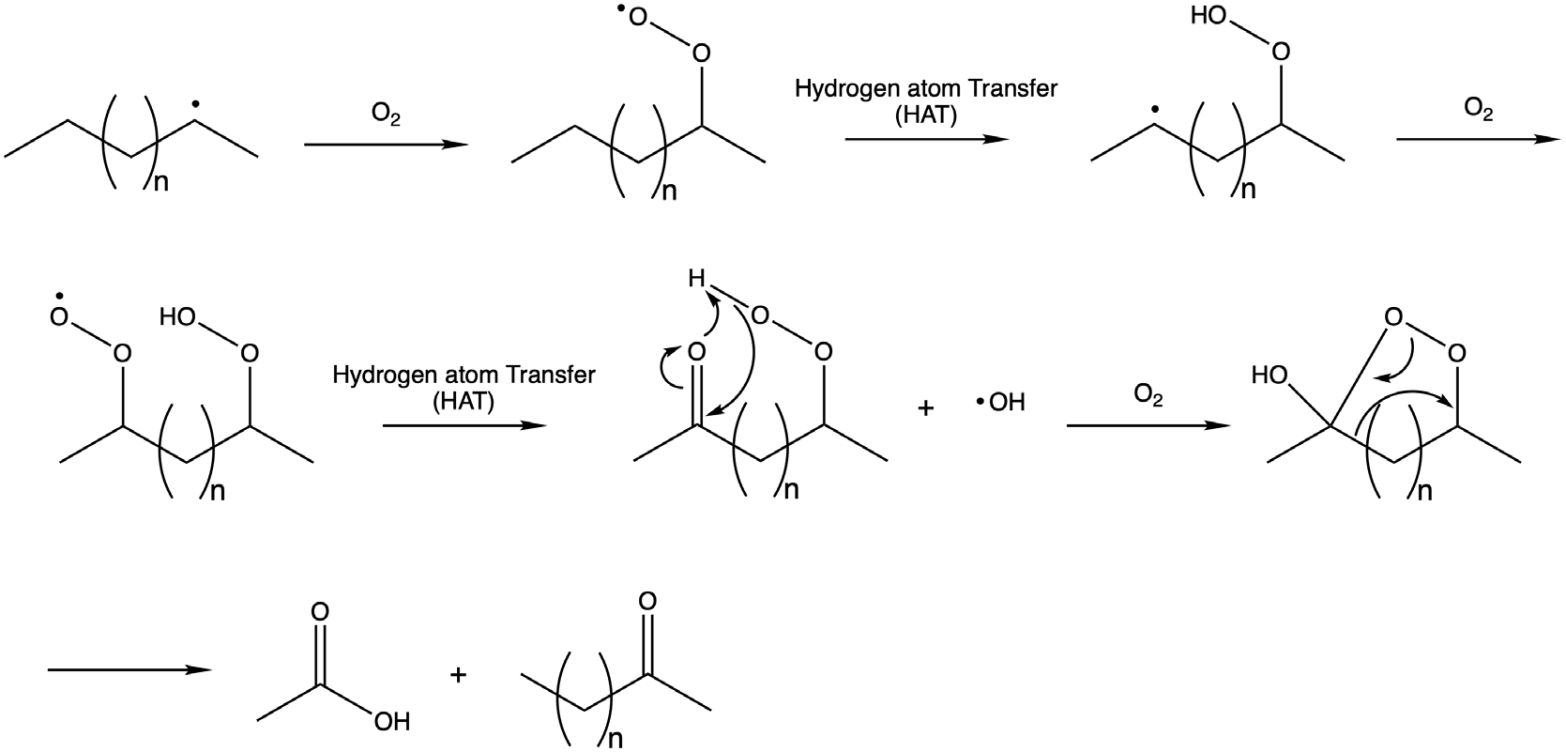
Proposed reaction scheme for the chain scission during the oxidation reaction.

## 5 Conclusions

AIBN and carboxylic acids such as HOAc can promote aerobic oxidation of long‐chain hydrocarbons by generating radicals that initiate an autoxidation chain reaction sequence. The observations obtained from the model compound C_20_ translate effectively to polymer systems, reinforcing the general applicability of the observed trends. While some polymers already have a certain degree of intrinsic oxidation and the viscosity in the molten state can weaken the efficiency of radical initiators, the overall oxidation behavior remains consistent. However, this enhancement is accompanied by undesirable C─C bond scission, leading to a reduction in molecular weight. Lowering the oxygen partial pressure can partially mitigate this effect, but a correlation persists between the extent of conversion and molecular weight degradation. These findings highlight a fundamental trade‐off between oxidation efficiency and polymer backbone integrity in the autoxidation of polymer substrates.

While AIBN has been used extensively as an initiator for radical chain reactions, the use of HOAc in this role is unusual. HOAc is proposed to similarly promote oxidation through a radical chain pathway with minimal chain scission when oxygen partial pressure is controlled. The presence of a carboxylate group or at least a carbonyl group (see results with acetaldehyde) appears to be critical for this reactivity. Noncarboxylic acids did not promote autoxidation. Challenges arise as greater amounts of oxygen are incorporated into the backbone of the polymer with either AIBN or HOAc, as further reaction of oxygenated polymer with oxygen promotes chain degradation.

Overall, it was determined that AIBN and HOAc are both effective as radical initiators for hydrocarbon and polymer autoxidation reactions. With proper control of temperature and oxygen pressure, C─C scission can be minimized as long as oxygen incorporation into the backbone is kept at low levels. The reactivity trends observed for the model alkane *n*‐eicosane translated to PE4k, affirming that mechanistic insights derived from small‐molecule systems can inform polymer oxidation behavior. The findings reported here provide a framework for using inexpensive and readily available initiators for polymer autoxidation. The operating temperature and pressure are critical levers in achieving mild functionalization, enhanced oxygen incorporation, and controlled molecular weight reduction for polymer upcycling applications.

## Funding

This study was supported by U.S. Department of Energy (DE‐SC0022238).

## Conflicts of Interest

The authors declare no conflicts of interest.

## Supporting information

Supplementary Material

## Data Availability

The data that support the findings of this study are available from the corresponding author upon reasonable request.
