# Supplementary material for: Controlled Oxidation of n‐Eicosane and Low‐Molecular‐Weight Polyethylene
Source: ChemSusChem. 2026 Oct 12;19(19):e71130. doi: 10.1002/cssc.71130 (PMC13655978; doi:10.1002/cssc.71130)
Supplement: Supplementary file 1 [file CSSC-19-e71130-s001.pdf]

Supplementary Materials

Controlled Oxidation of *n*-Eicosane and Low Molecular Weight Polyethylene

Zhuoming Feng[a]†, Maura Gibbs[b]†, Anaise Thomas[b], Pedro Jimenez Antenucci[b], Hoda Shokrollahzadeh Behbahani[c], Karen I. Winey[a,c], Marisa C. Kozlowski[b], Karen I. Goldberg[b]*, and John M. Vohs[a]*

[a] Department of Chemical and Biomolecular Engineering, University of Pennsylvania, Philadelphia, Pennsylvania 19104, USA

[b] Department of Chemistry, University of Pennsylvania, Philadelphia, Pennsylvania 19104, USA

[c] Department of Materials Science and Engineering, University of Pennsylvania, Philadelphia, Pennsylvania 19104, USA

† These authors contributed equally.

Table of Contents

I. General Considerations S2
II. General Reaction Procedures for Oxidation Reactions S3
III. Eicosane ($C_{20}H_{42}$) Products Characterization Data S5
IV. PE4k Products Characterization Data S12
V. Theoretical Calculation of product conversion S14
VI. References S16

List of Substrates and Lot #s:

n-eicosane ($C_{20}H_{42}$): Sigma-Aldrich (99%) | LOT #MKCR8596
n-eicosane ($C_{20}H_{42}$): Acros (99%) | LOT #8ISXB-J
Polyethylene: (M_w 4,000/M_n 1,700): Sigma Aldrich | LOT # MKCW2074
Polyethylene: (M_w 4,000/M_n 1,700): Sigma Aldrich | LOT #MKCL2659

General Considerations

Materials

Alkane (n-eicosane) and PE (polyethylene) substrates were purchased and used without further purification. Deuterated $CDCl_3$ used to analyze alkane dehydrogenation reaction samples at room temperature was purchased from Cambridge Isotope Labs. For ^{1}H-NMR spectroscopy of polymer samples at elevated temperatures, tetrachloroethane-d_2 was used as purchased from Cambridge Isotope Labs. Acetic acid (Glacial, ACS Grade) was purchased from Fisher Scientific. Radical initiator 2,2'-Azobis(2-methylpropionitrile) (AIBN), and 1,1'-Azobis(cyclohexane carbonitrile) (ABCN) was purchased from Sigma-Aldrich (98%) were used without further purification. The n-eicosane products were analyzed via ^{1}H-NMR, FTIR, and GC-MS. The polyethylene products were analyzed by ^{1}H-NMR (high temp), FTIR, and GPC. Instrument details provided below.

^{1}H-NMR Characterization

The ^{1}H-NMR spectroscopy of eicosane and PE4k samples were performed in 5mm diameter tubes on a Bruker 500 spectrometer at 500 MHz. The ^{1}H-NMR spectroscopy of eicosane was collected in chloroform-*d* at ambient probe temperatures. The ^{1}H-NMR spectroscopy of PE4k were performed in 1,1,2,2-tetrachloroethane-d_2 between 60 °C–80 °C. ^{1}H-NMR spectral data are reported in ppm and were referenced to residual protons in the deuterated solvents: chloroform-*d* (7.26 ppm) and TCE-d_2 (1.94 ppm). Spectra used for integration were acquired using 128 scans, a relaxation delay (D1) of 3.0 s, and a spectral width of 20 ppm.

T_1 values of the PE4k backbone as well as ketone and alcohol-containing resonances were not independently measured in this study. As such, the ^{1}H-NMR integrations are used as relative and semiquantitative estimates of CH_2 conversion and product distribution. The consistency of these ^{1}H-NMR derived conversions is supported by comparison with the obtained GC-MS data for C_{20}. For the C_{20} reactions analyzed by both methods, the CH_2 conversions estimated by ^{1}H-NMR and GC-MS were in close agreement. For example, oxidative conversions of 0.5% and 0.5% were obtained by GC-MS and ^{1}H-NMR, respectively, for reaction at 90 °C with 50 mg AIBN for 10 h, while values of 0.9% and 0.9%, respectively, were obtained for reaction at 110 °C with 50 mg AIBN for 5 h.

For PE4k, ^{1}H-NMR was used to compare the relative extent of functionalization between samples because direct GC-MS analysis is not applicable to the polymer. Literature measurements can provide some useful context for the expected ^{1}H spin–lattice relaxation times (T_1) of polyethylene. For example, Packer an coworkers reported ^{1}H T_1 values of approximately 1.3–2.6 s for semi-crystalline polyethylene samples.[1] These measurements were obtained under conditions different from the solution-state, elevated-temperature measurements used in the present study and therefore are not assumed to represent the exact T_1 values of the resonances analyzed here.

GC-MS Characterization

The GC-MS of C_{20} was collected using a gas chromatography-mass spectrometry (Shimadzu model QP-5000 GC-MS) equipped with a capillary column (HP-5, 19091J-433, Agilent Technologies, Inc.). The solid products obtained for reaction of C_{20} were collected and dissolved in toluene (ACS Grade) in a 1-to-10 vol ratio.

HT GPC-IR Characterization

Molecular weight and dispersity (Đ) were determined by high temperature gel permeation chromatography system with infrared detection (HT GPC-IR, Polymer Char, IR4 detector) and analyzed using GPC One software (v3.3). The detector monitors total polymer concentration and methyl-specific signals in the region of 2750–3000 cm^{-1}. The mobile phase was 1,2,4-$C_6H_3Cl_3$ (TCB) with 300 ppm BHT at 1.0 mL min^{-1}. Separation used a PLgel Olexis column set (Agilent, 13 µm): a guard column (7.5 × 50 mm) and three analytical columns (7.5 × 300 mm) in series. Injector, columns, and detector were held at 150 °C. Crimp-sealed vials were purged with N_2 (3×) before heating. Samples (1 mg mL^{-1}) were dissolved in TCB at 150 °C for 100 min, passed through a 10 µm inline stainless-steel filter, and injected immediately after dissolution. The concentration signal was calibrated with 15 narrow-dispersity polystyrene standards (Polymer

Char PMCP-00018201); data were processed with GPC One software (v3.3). Mark–Houwink parameters for polystyrene-to-polyethylene conversion were α = 0.722, k = 0.01016 mL/g (PS); α = 0.725, k = 0.04416 mL/g (HDPE).[2] The CH_3 signal was calibrated with five ethylene-1-octene copolymer standards (2–36 wt% comonomer; Polymer Char PMCP-000184). Short chain branching is reported as CH_3 per 1000 carbons (CH_3/1000C) and the IR4 composition detection limit is ~10 CH_3/1000C.

FTIR Characterization:

Nicolet iS50 FTIR spectrometer equipped with an attenuated total reflectance (ATR) module. Solid products samples were compressed against a diamond ATR crystal using an anvil. ATR-FTIR spectra were recorded with a spectral resolution of 0.2 cm^{-1}, and each spectrum was obtained by averaging 100 scans.

Batch Reactor:

Parr 5000 six-reactor high-pressure system (30 mL volume) (Figure S1b). 5000 Multi Reactor (5000 six-reactor) Heater and Stirrer System with a 4871 Process Controller. Each individual stirred vessel can operate up to 3000 psi and 300 °C. The heat can be monitored by the well or an internal reactor thermocouple.

Batch Pressure Reactor:

Oxidation reactions using AIBN were conducted in a temperature and pressure controlled stainless steel Ollital Pressure Vessel (model OLT-HP-50) (Figure S1a). The reactor internal volume was 50mL and temperature is controlled via an internal reactor thermocouple.

General Reaction Procedures for Oxidation Reactions

The hydrocarbon reactants used in this study were *n*-eicosane ($C_{20}H_{42}$, 99%, Sigma-Aldrich), which will be referred as C_{20}, and polyethylene (average Mw ~4,000 Da by HT GPC-IR, average Mn ~1,700 Da by HT GPC-IR, Sigma-Aldrich) which will be referred to as PE4k. For the experiments conducted directly in the reactor vessel, reactions were performed using a 50 mL, temperature and pressure-controlled, stainless steel, magnetic stirred tank reactor (Ollital, model OLT-HP-50). For each run, 3g *n*-eicosane or PE4k were loaded into the reactor along with a specified amount of either AIBN or ABCN, which were used as radical initiators. Some reactions were also conducted without using an initiator. The reactor was then sealed and purged 5–6 times with either O_2 or He at 1 bar before pressurizing with one of these gases to the desired reaction pressure. The reactor was then heated to the reaction temperature for the desired reaction time while the liquid phase was stirred with a magnetic stir bar rotating at 200 rpm. At the end of the reaction, the reactor was rapidly quenched in an ice bath.

For reactions carried out with acetic acid, 1.5 g of substrate (C_{20} or PE4k) was added to Parr batch reactors with a 30 mL internal volume (Figure S1b). To minimize potential reaction of acid with the stainless-steel reactor, these reactions (both for C_{20} and PE4k) were conducted in a 20 mL borosilicate glass vial equipped with a magnetic stir bar. Acetic acid was added to reaction vial via syringe. The vials were placed in a Parr high pressure reactor vessel and the vessel was sealed and purged with the desired gas, pressurized to the specified pressure, and heated for the indicated reaction time. Following reactions containing HOAc, the recovered material was dried *in vacuo* prior to analysis to remove residual acetic acid.

Control experiments without added HOAc were also conducted to evaluate the extent of oxidation in the absence of acetic acid and the effect of reactor configuration. For PE4k, a control reaction was performed in a borosilicate vial at 125 °C under 0.1 MPa O_2 for 18 h without added HOAc. Additional controls without acetic acid were performed with PE4k placed directly in the stainless-steel reactor, without the borosilicate vial, at 125 °C under either 0.1 MPa O_2 for 18 h or 0.5 MPa O_2 for 4 h. These experiments correspond to Entries 3–5 of Table 7. Comparison of the reactions conducted in the glass vial and directly in the stainless-steel reactor was used to evaluate effect of reactor configuration independently of the added HOAc.

Note that the AIBN-initiated and HOAc-assisted PE4k oxidation experiments were performed in different laboratories using different batch reactor systems and different lots of PE4k. Checks were made to ensure consistency across the two reactor systems.

The solid products obtained for reaction of C_{20} were collected and dissolved in toluene in a 1-to-10 vol ratio, and then the composition of the solution was determined using a gas chromatography-mass spectrometry (GC-MS). The solid products when using PE4k as the reactant and some C_{20} solid products were collected and characterized using ^1H nuclear magnetic resonance (^1H-NMR) spectroscopy to determine the type and position of the functional groups. Fourier-Transform Infrared Spectroscopy (FTIR) was also used to characterize reaction products.

a.

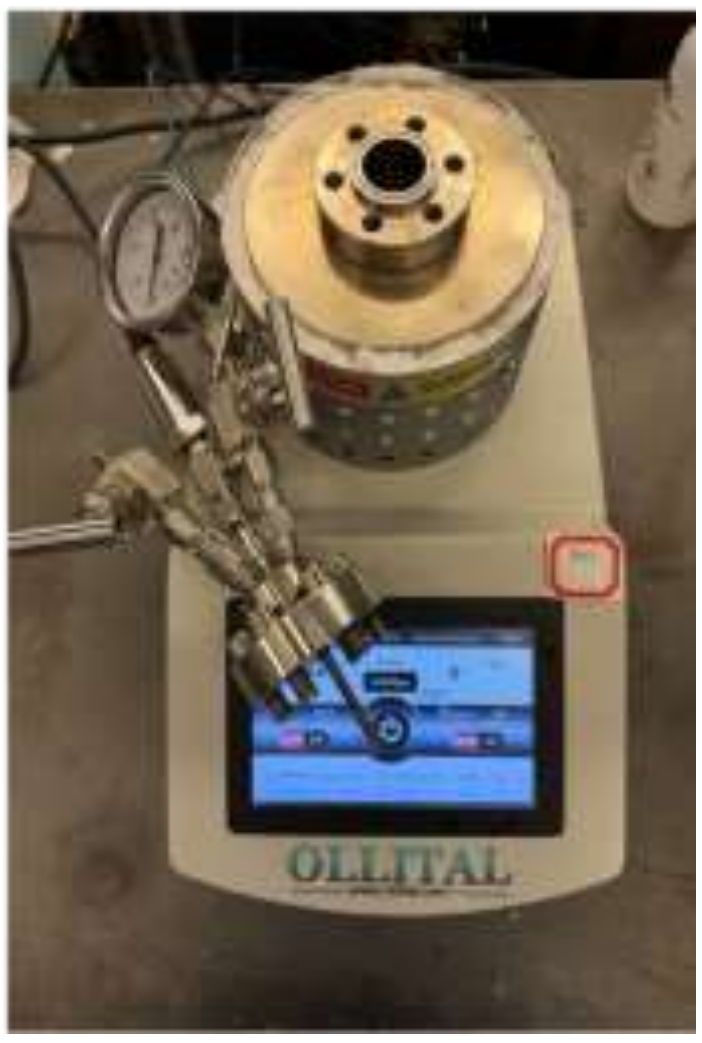

b.

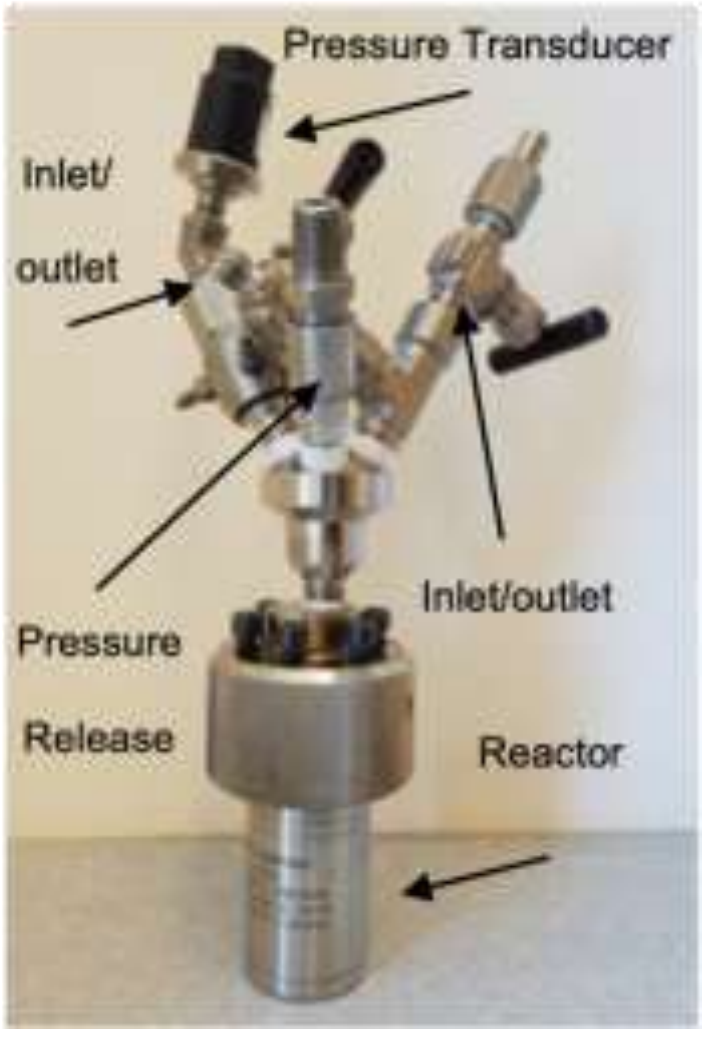

Figure S1a. Picture of the components of Ollital batch Reactor, comprised of 50 mL stainless steel reactor vessel and a lid with temperature sensor connector and pressure gauge.

Figure S1b. Picture of the components of Parr Reactor, comprised of stainless steel, with a lower accuracy pressure transducer (error +/- 2 bar), and a 30 mL reactor volume.

Eicosane ($C_{20}H_{42}$) Products Characterization Data

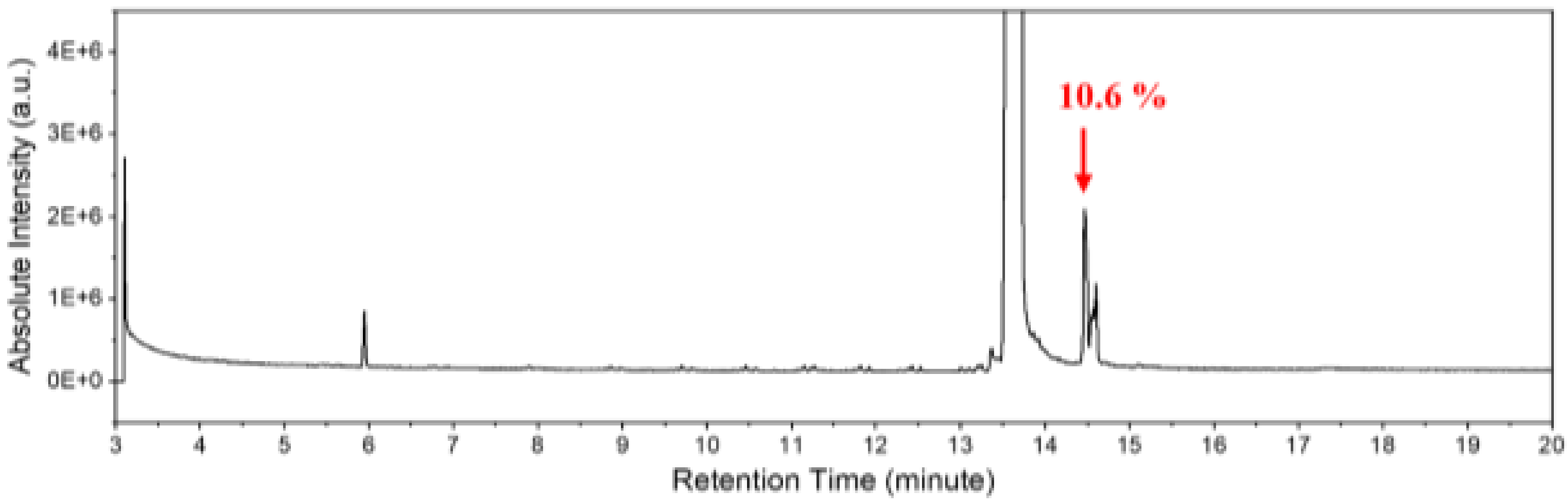

Figure S2. GC-MS trace of the C_{20} products for 3 g C_{20} under 90 °C 50 mg AIBN 0.5 MPa O_2, for 10 hours reaction.

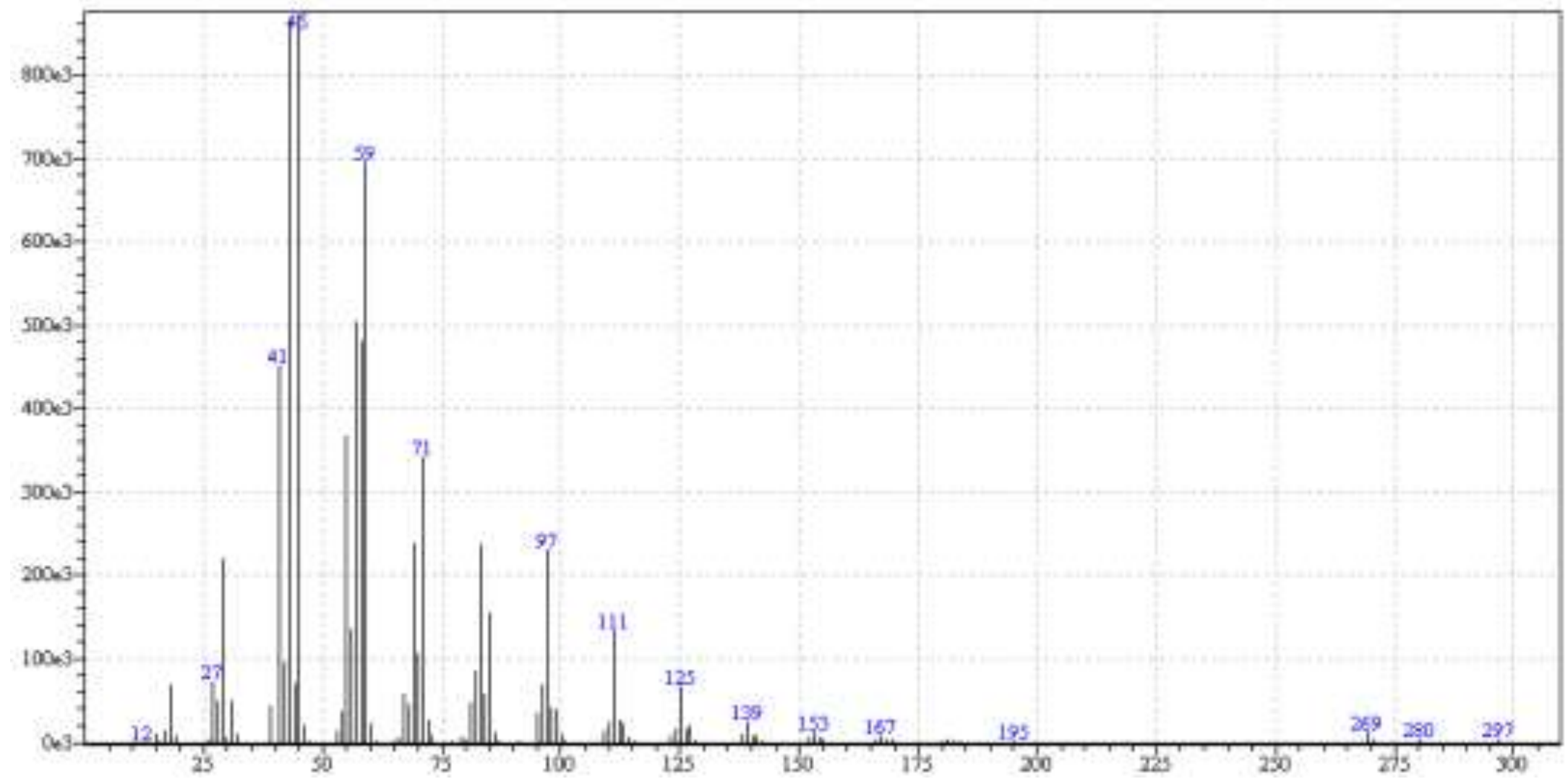

Figure S3. Mass spectrum electron ionization fraction of the C_{20} products C_{20}-ol.

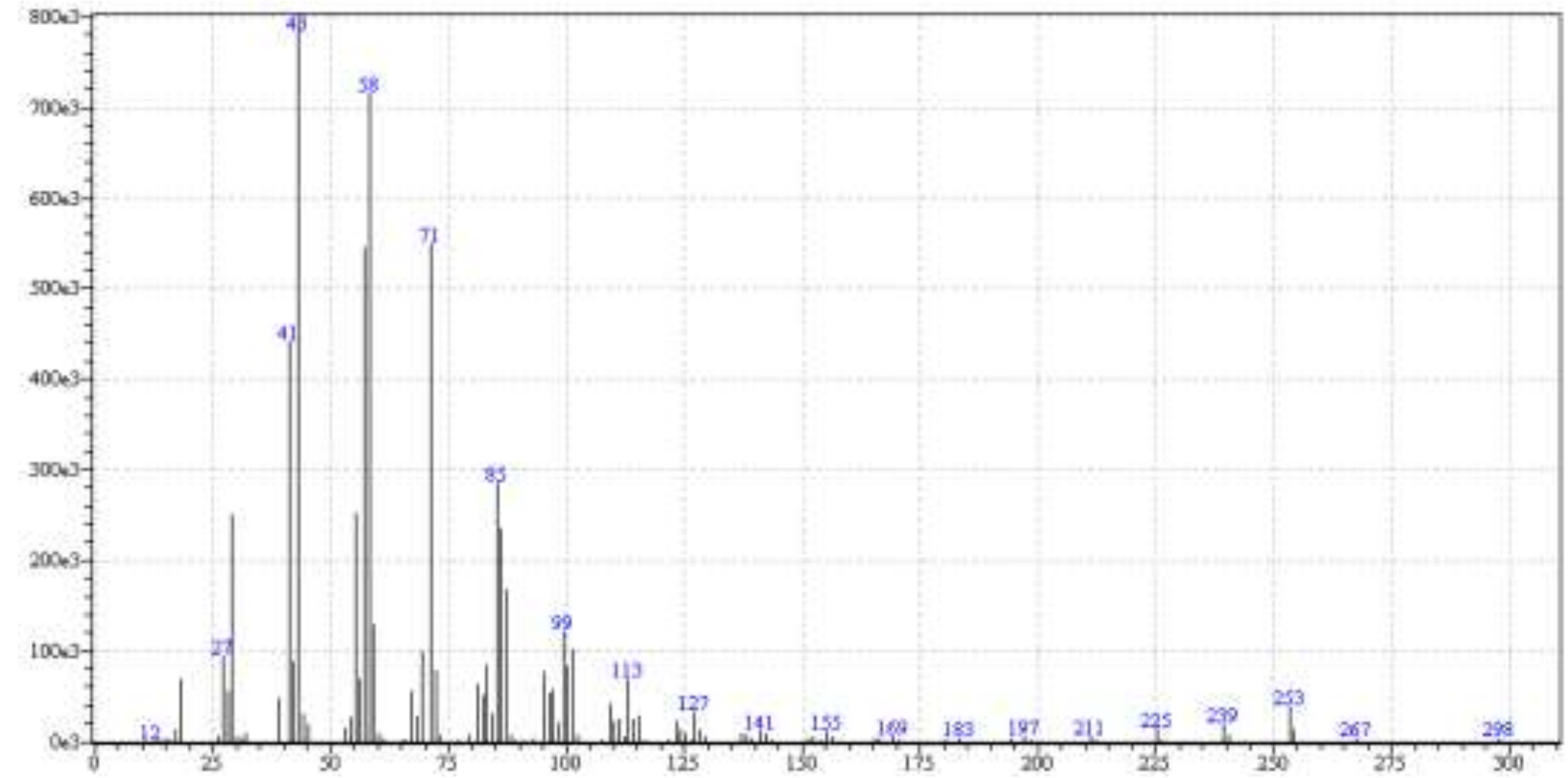

Figure S4. Mass spectrum electron ionization fraction of the C_{20} products C_{20}-one.

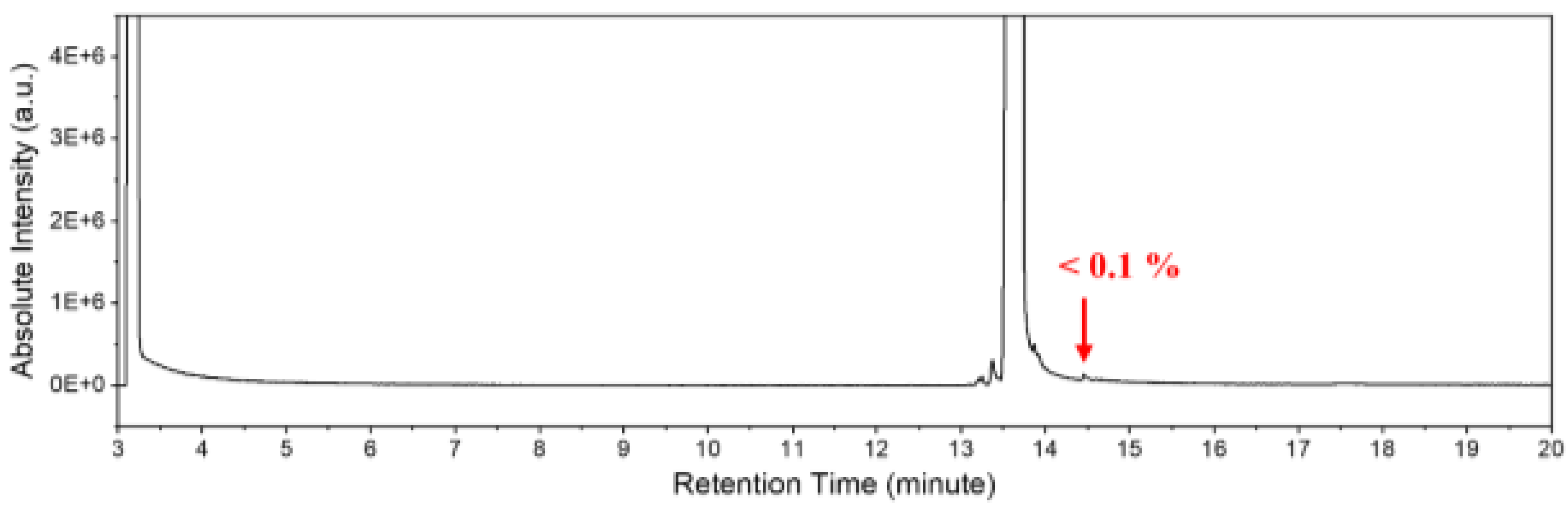

Figure S5. GC-MS trace of the C_{20} products for 3g C_{20} under 90℃, 0 mg AIBN 0.5 MPa O_2, for 10 hours reaction.

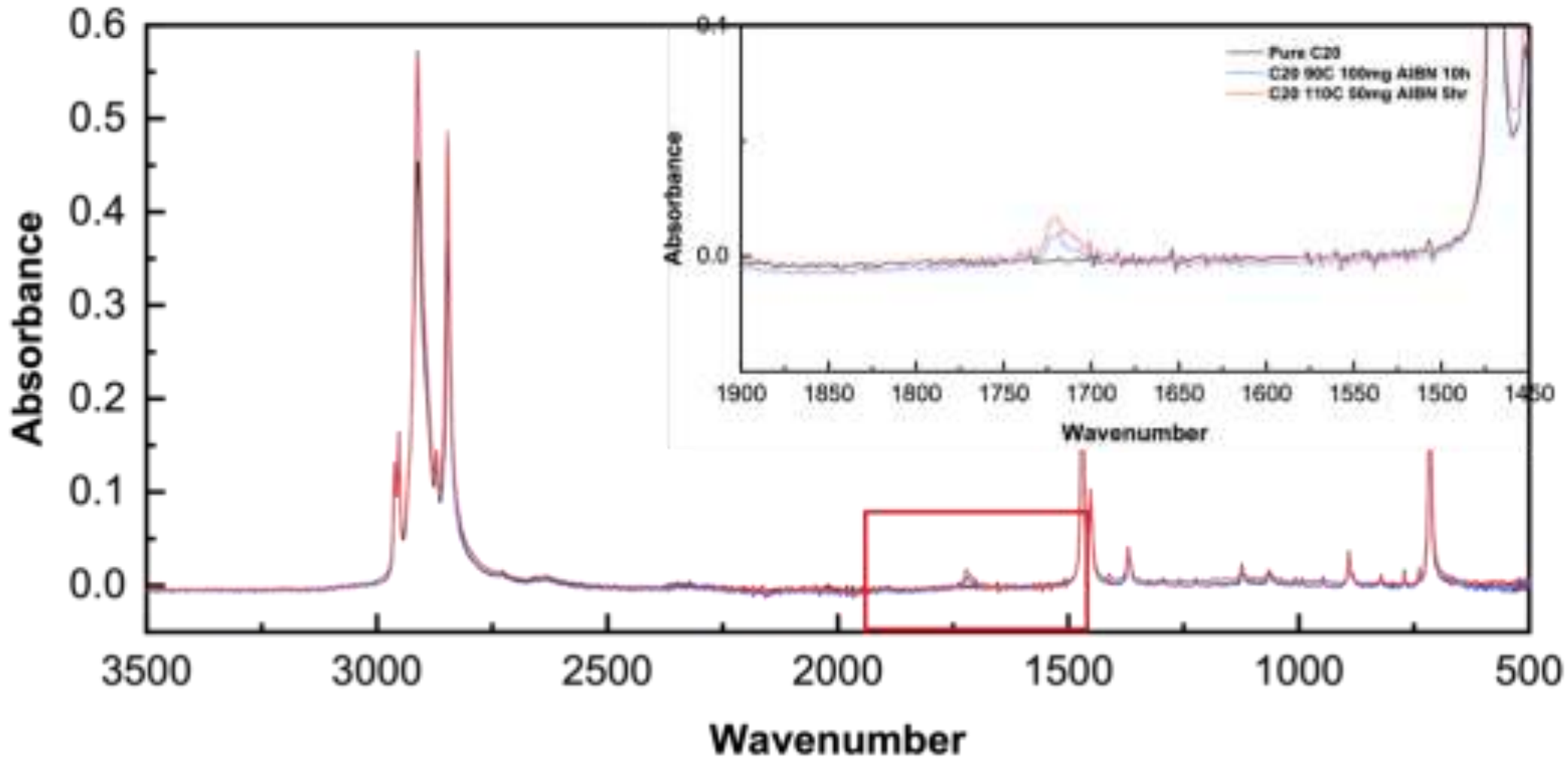

Figure S6. FTIR spectrum of pristine C_{20}, C_{20} reacted at 90 °C with 100 mg AIBN for 10 hours and C_{20} reacted at 110 °C with 50 mg AIBN for 5 hours.

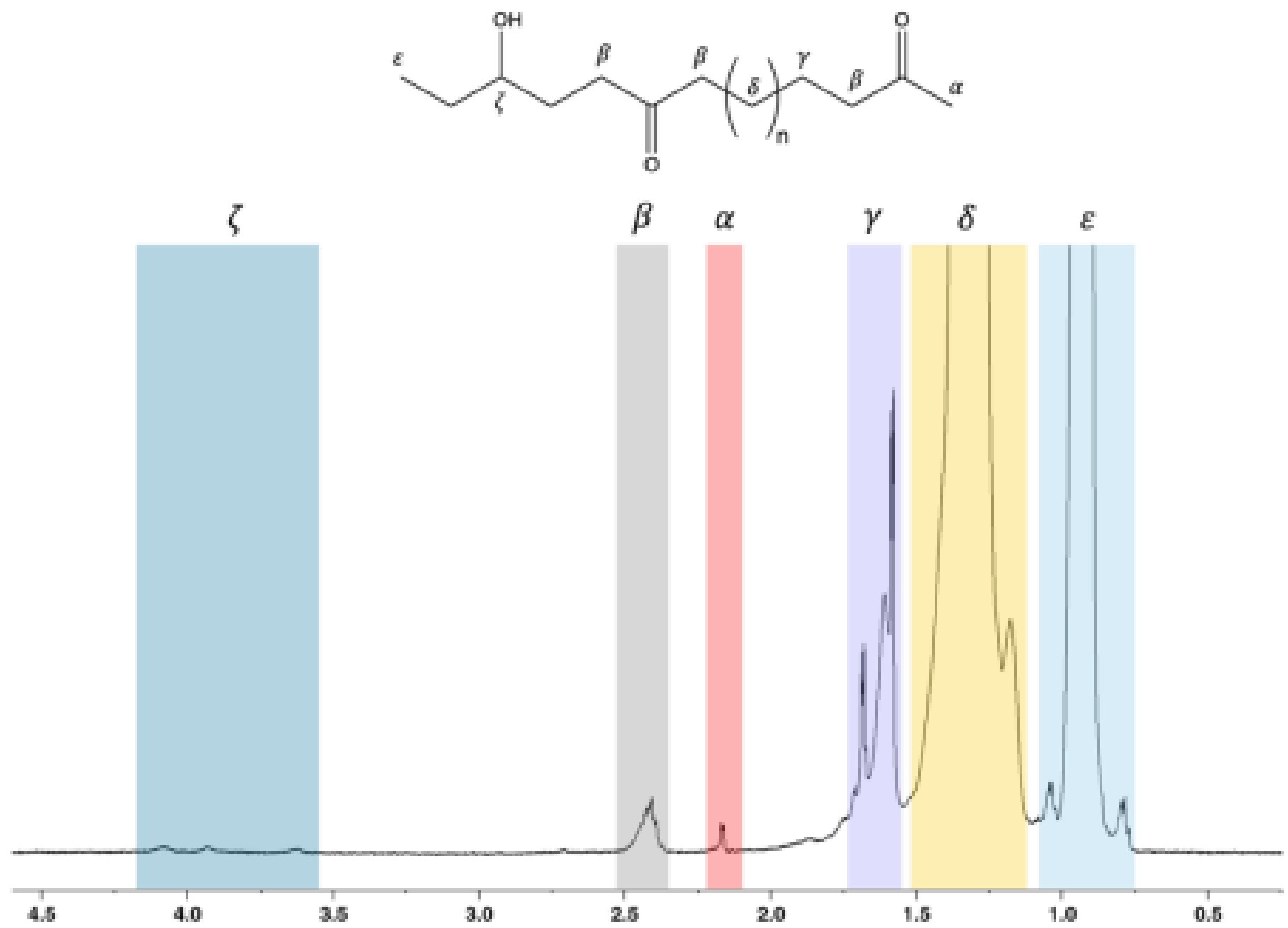

Figure S7. ^{1}H-NMR spectrum of C_{20} reacted at 90 °C with 100 mg AIBN for 10 hours.

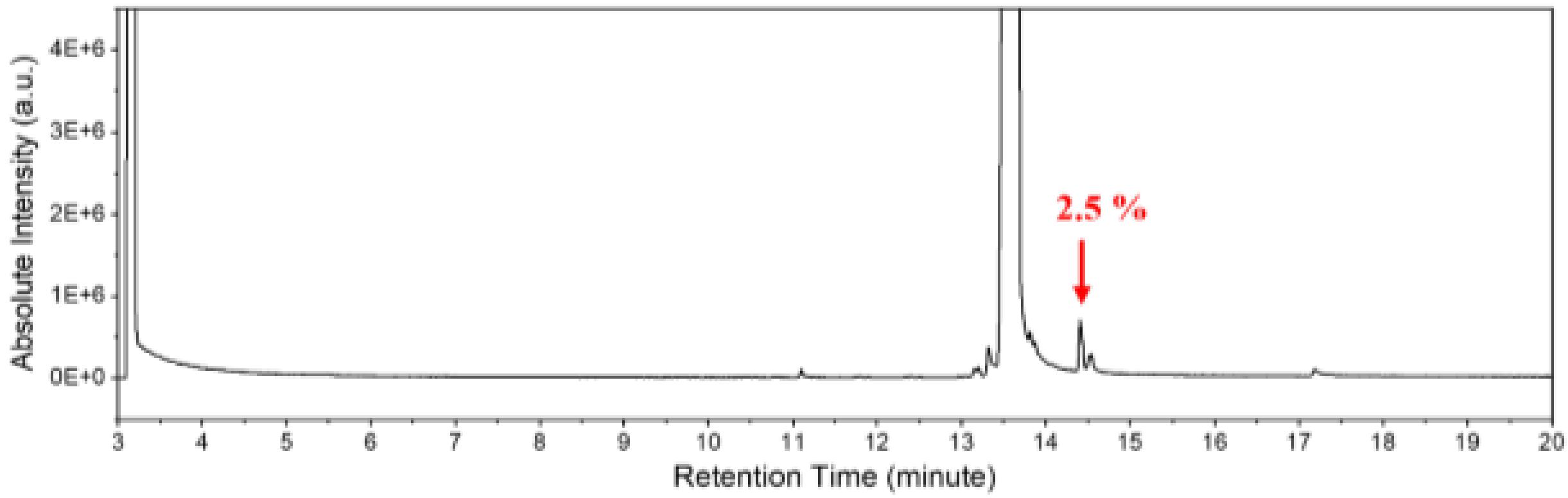

Figure S8. GC-MS trace of the C_{20} products for 3 g C_{20} under 110 °C 0mg AIBN 0.5 MPa O_2, for 5 hours reaction.

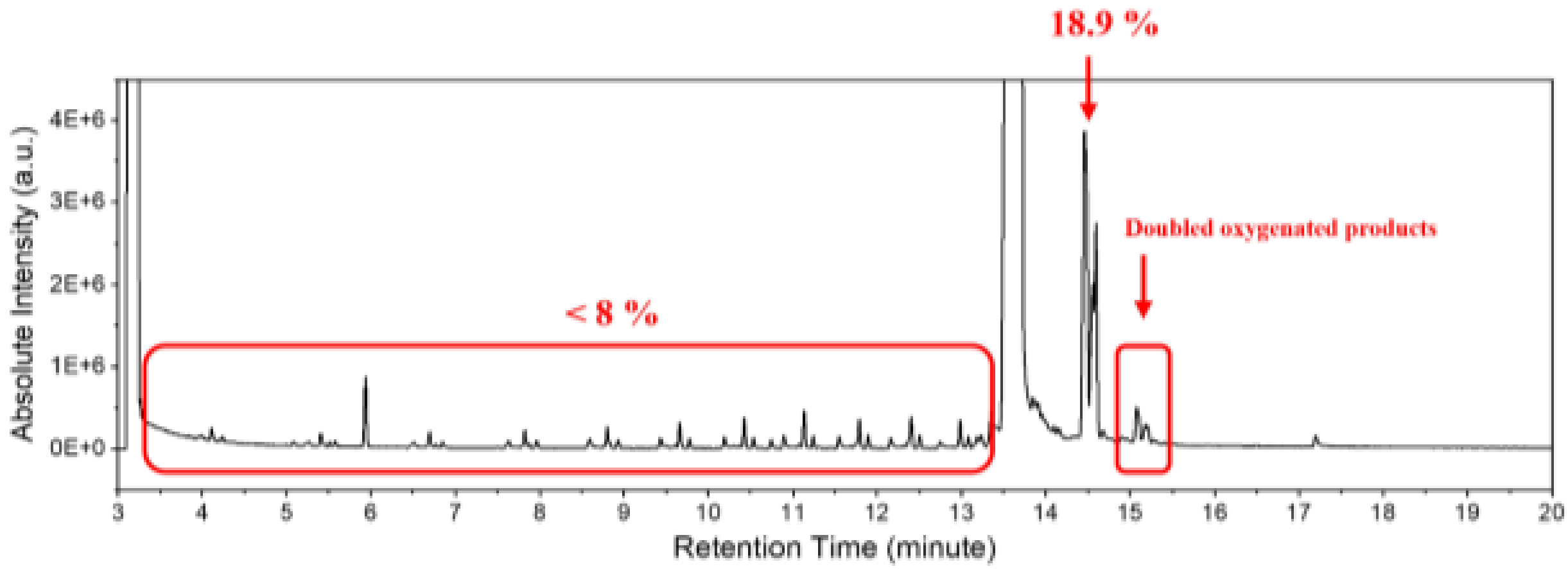

Figure S9. GC-MS trace of the C_{20} products for 3 g C_{20} under 110 °C 50 mg AIBN 0.5 MPa O_2, for 5 hours reaction. There are <8% small hydrocarbon derivatives products, and small amount doubled oxygenated products.

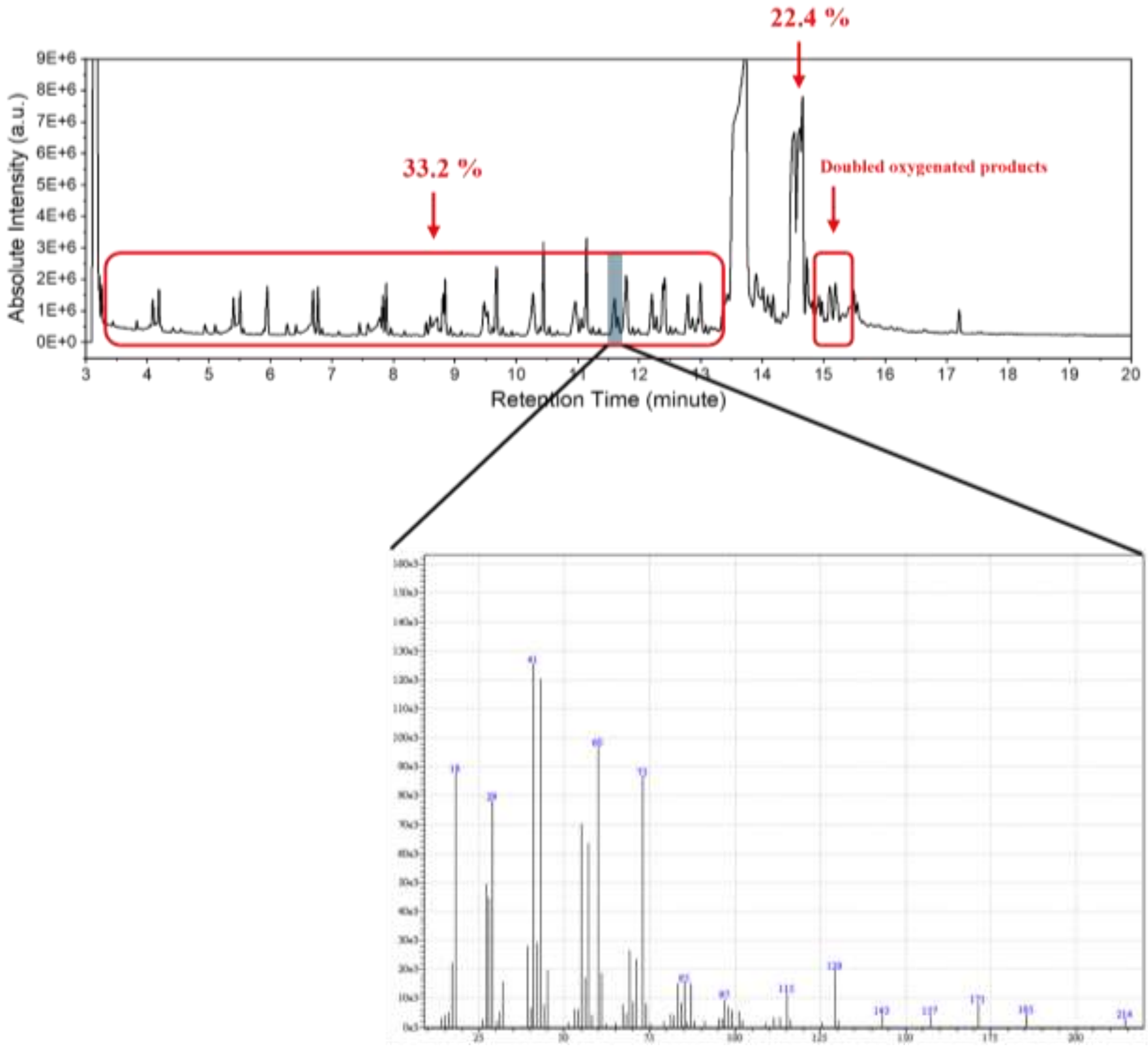

Figure S10. GC-MS trace and mass spectrum of the C_{20} products for 3 g C_{20} under 125°C 50 mg AIBN 0.5 MPa O_2, for 1 hour reaction. There are 33.2% small hydrocarbon derivatives, and 2.3% double oxygenated products.

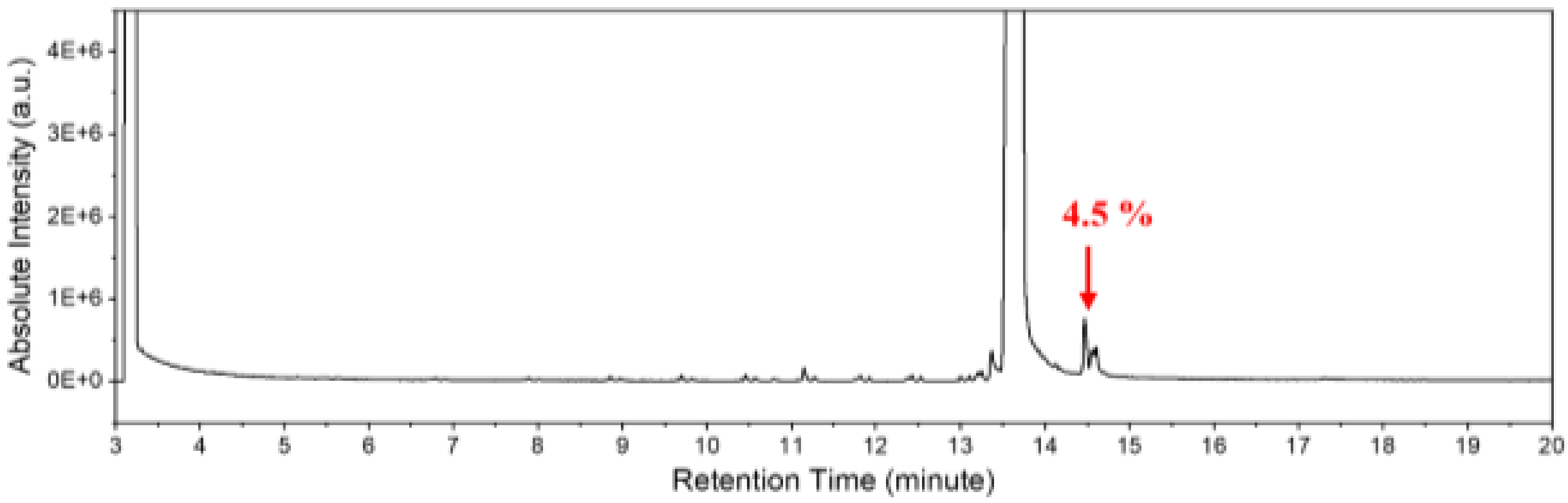

Figure S11. GC-MS trace of C_{20} reaction at 125 °C with HOAc (2.5 mol%) at 0.1 MPa O_2 for 18 hours.

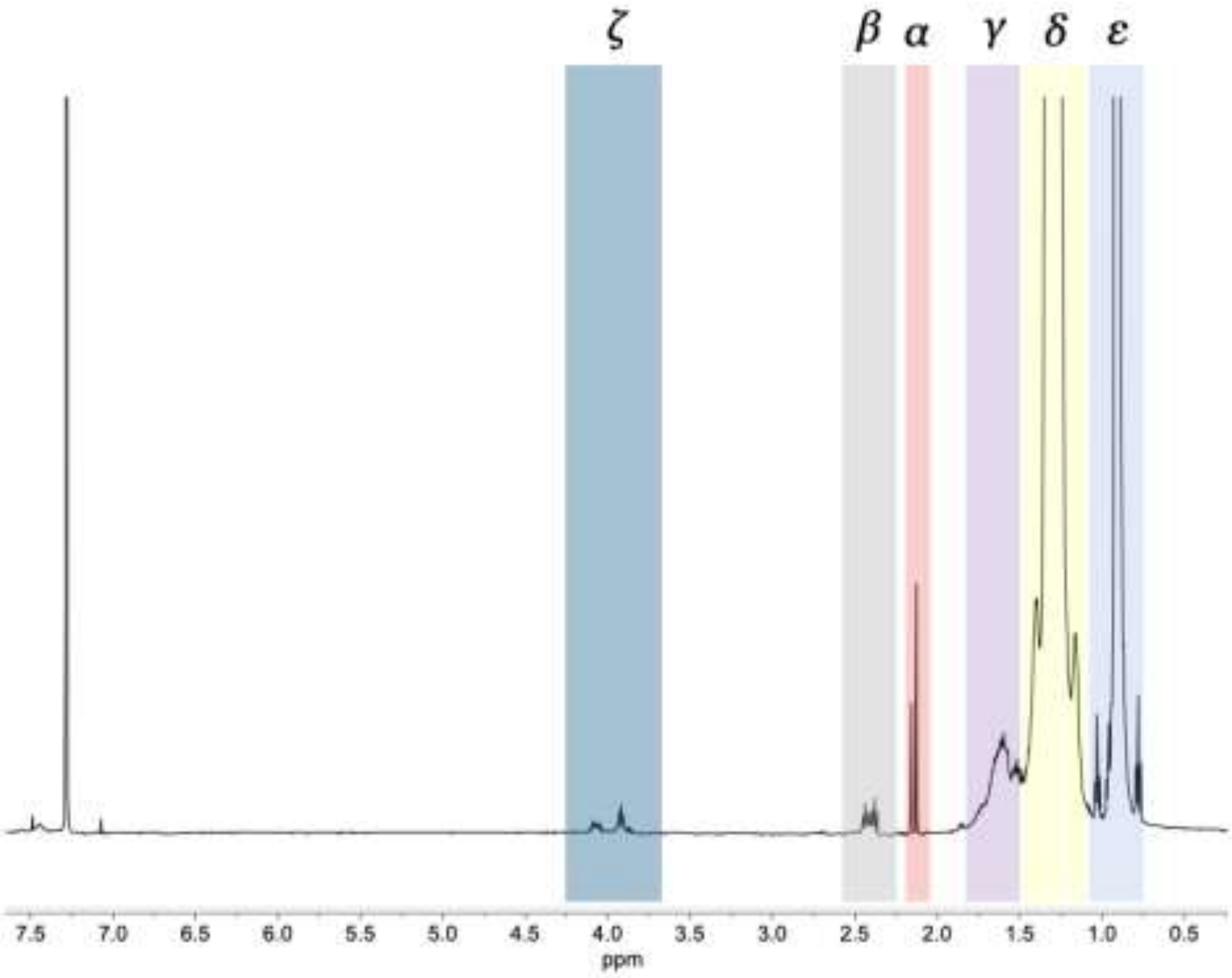

Figure S12. ^{1}H-NMR spectrum of C_{20} reaction at 125 °C with HOAc (2.5 mol%) at 0.1 MPa O_2 for 18 hours.

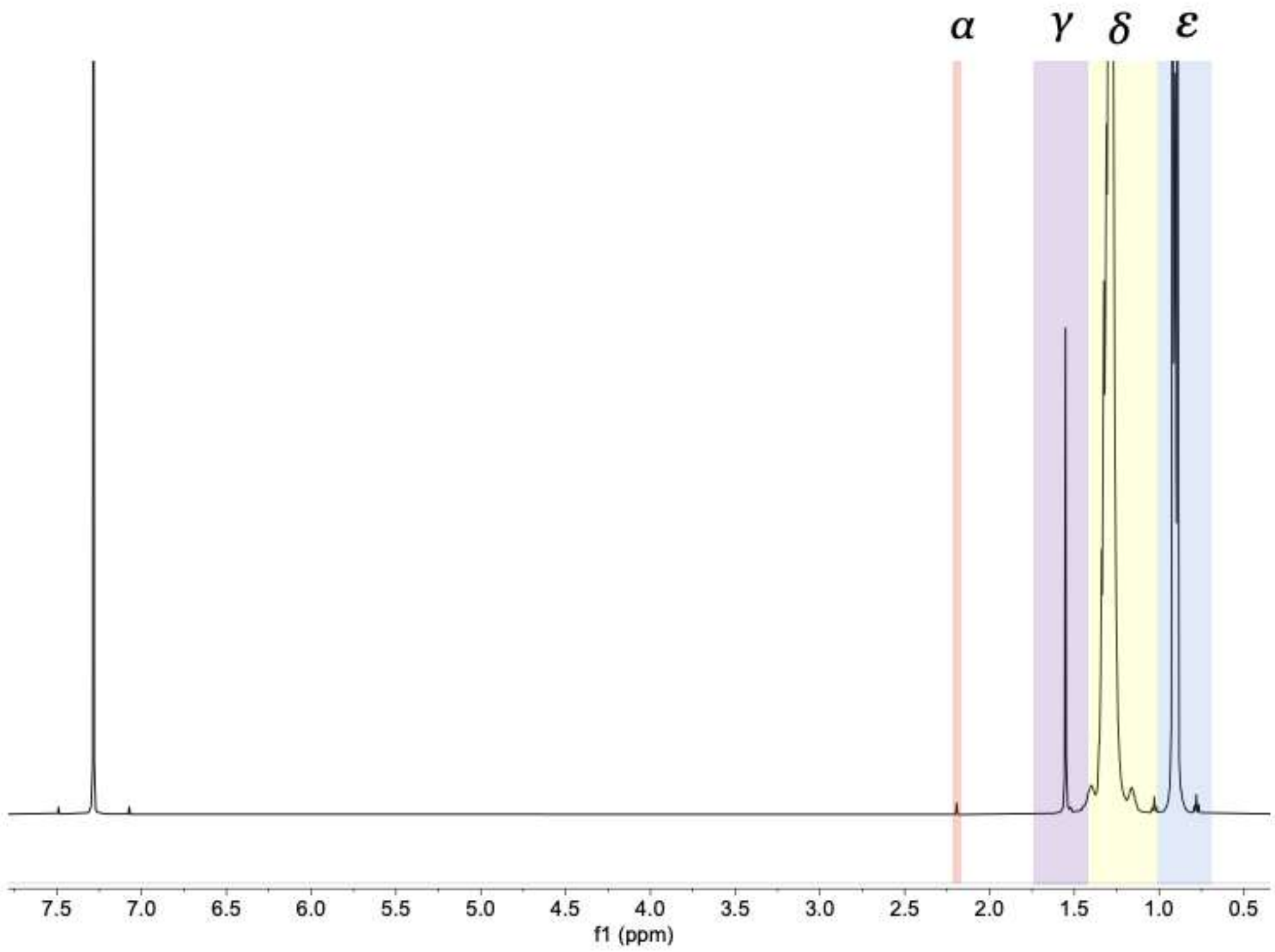

Figure S13. ^{1}H-NMR spectrum of C_{20} reaction at 125 °C without HOAc at 0.1 MPa O_2 for 18 hours.

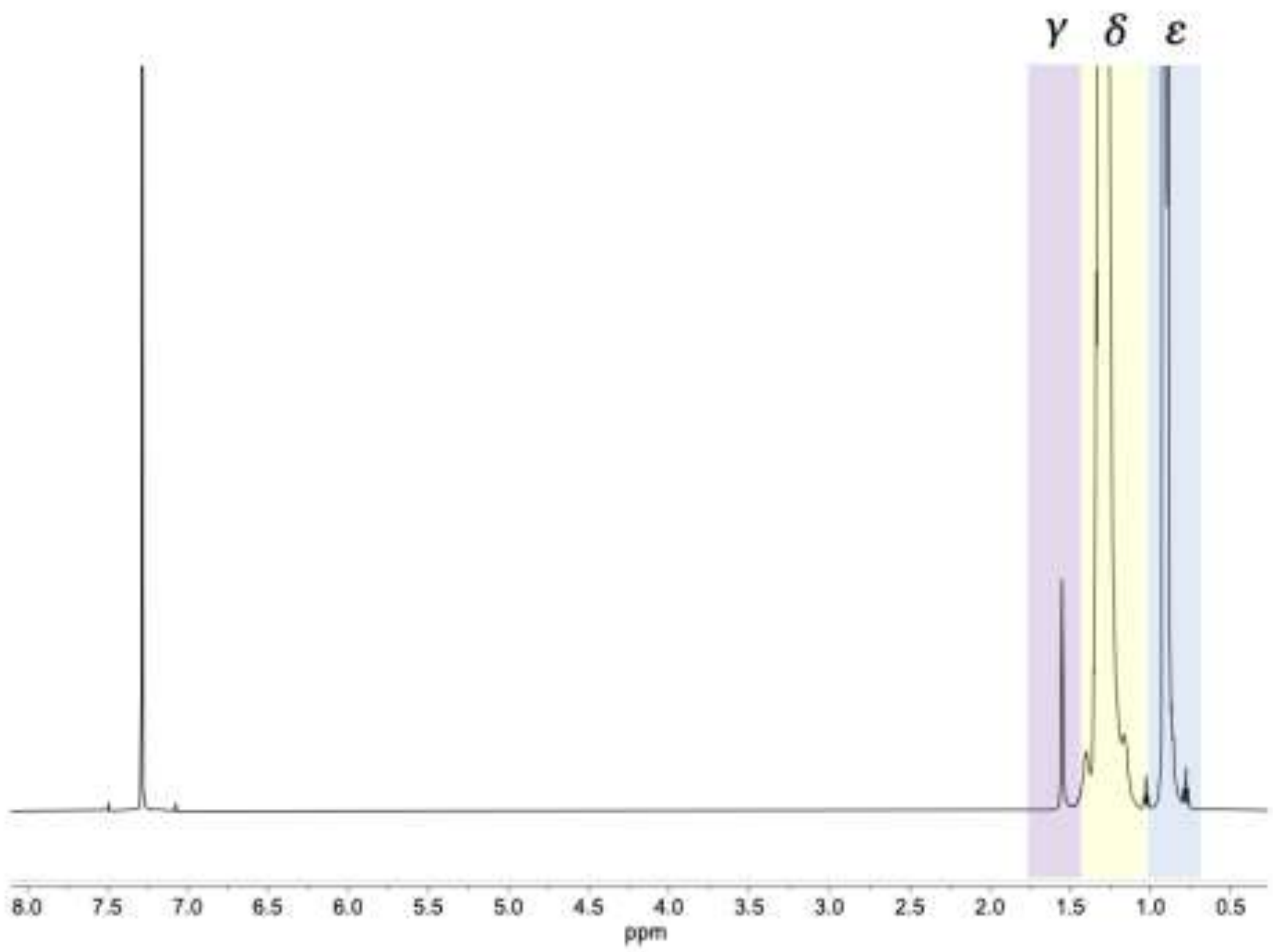

Figure S14. ^{1}H-NMR spectrum of C_{20} reaction at 125 °C with HOAc (2.5 mol%) at 0.1 MPa N_2 for 18 hours.

PE4k Products Characterization Data

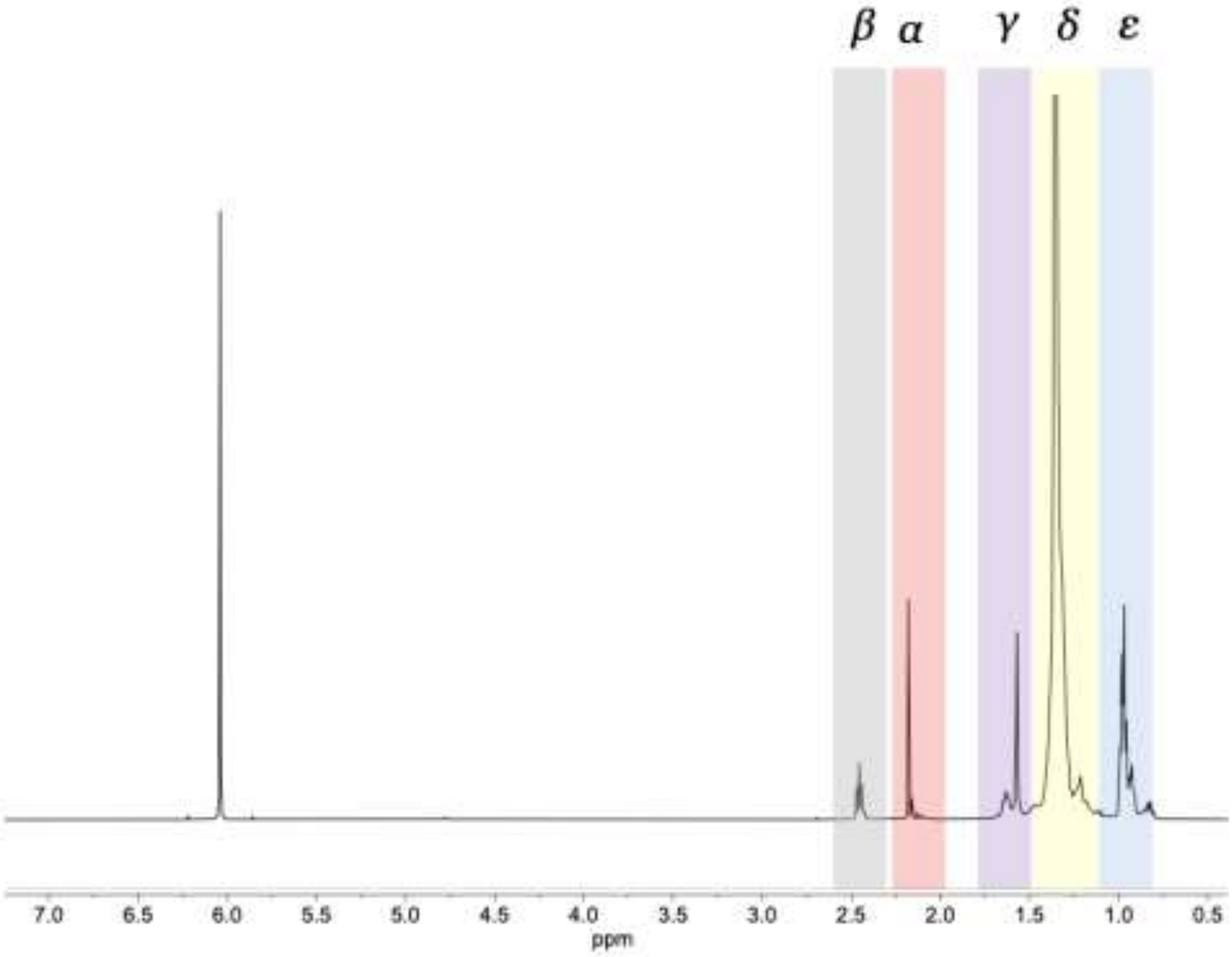

Figure S15. ^{1}H-NMR spectrum of base PE4k showing some initial oxidation in the backbone.

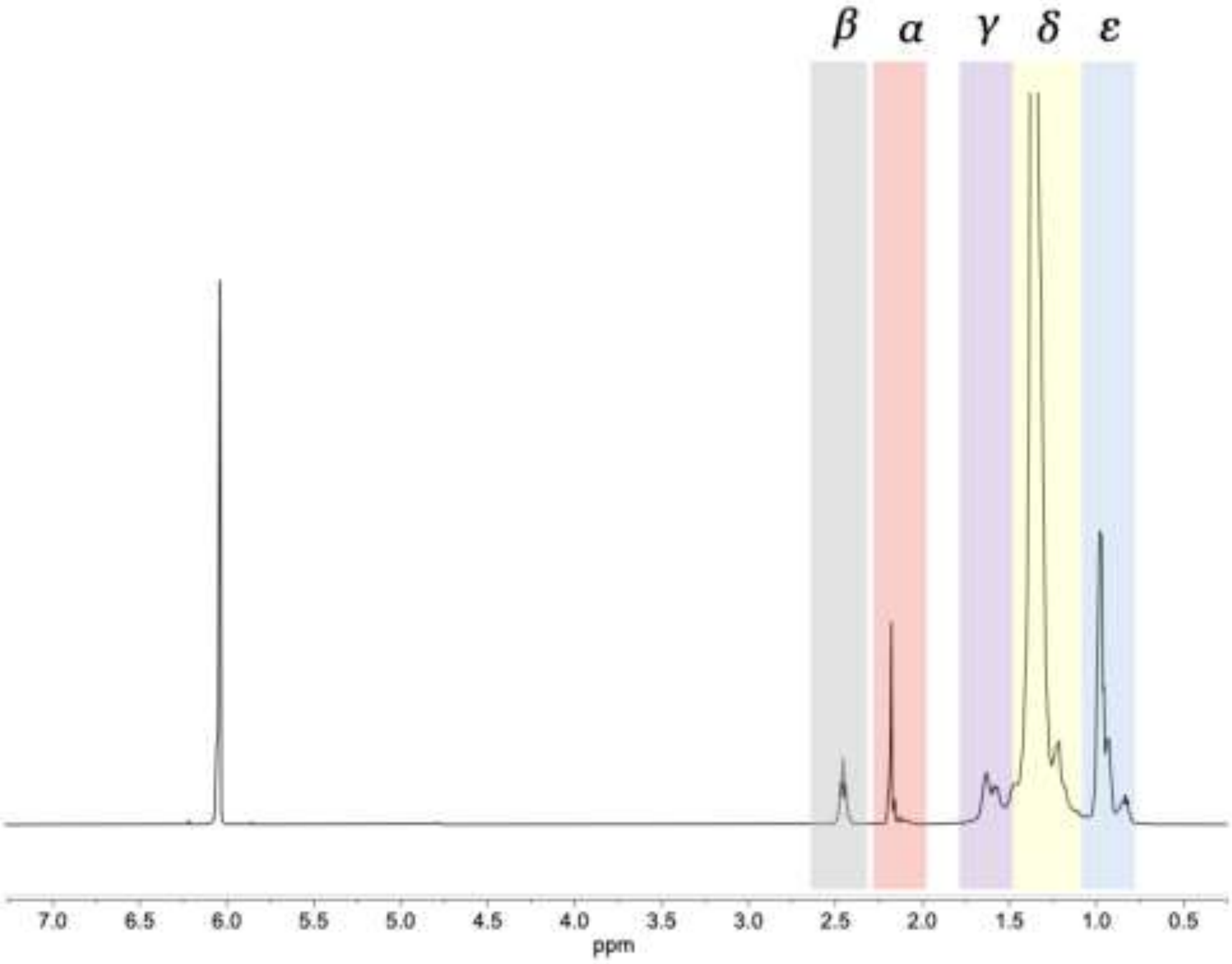

Figure S16. ^{1}H-NMR spectrum of PE4k reaction at 125 °C with HOAc (2.5 mol%) at 0.1 MPa N_2 for 18 hours.

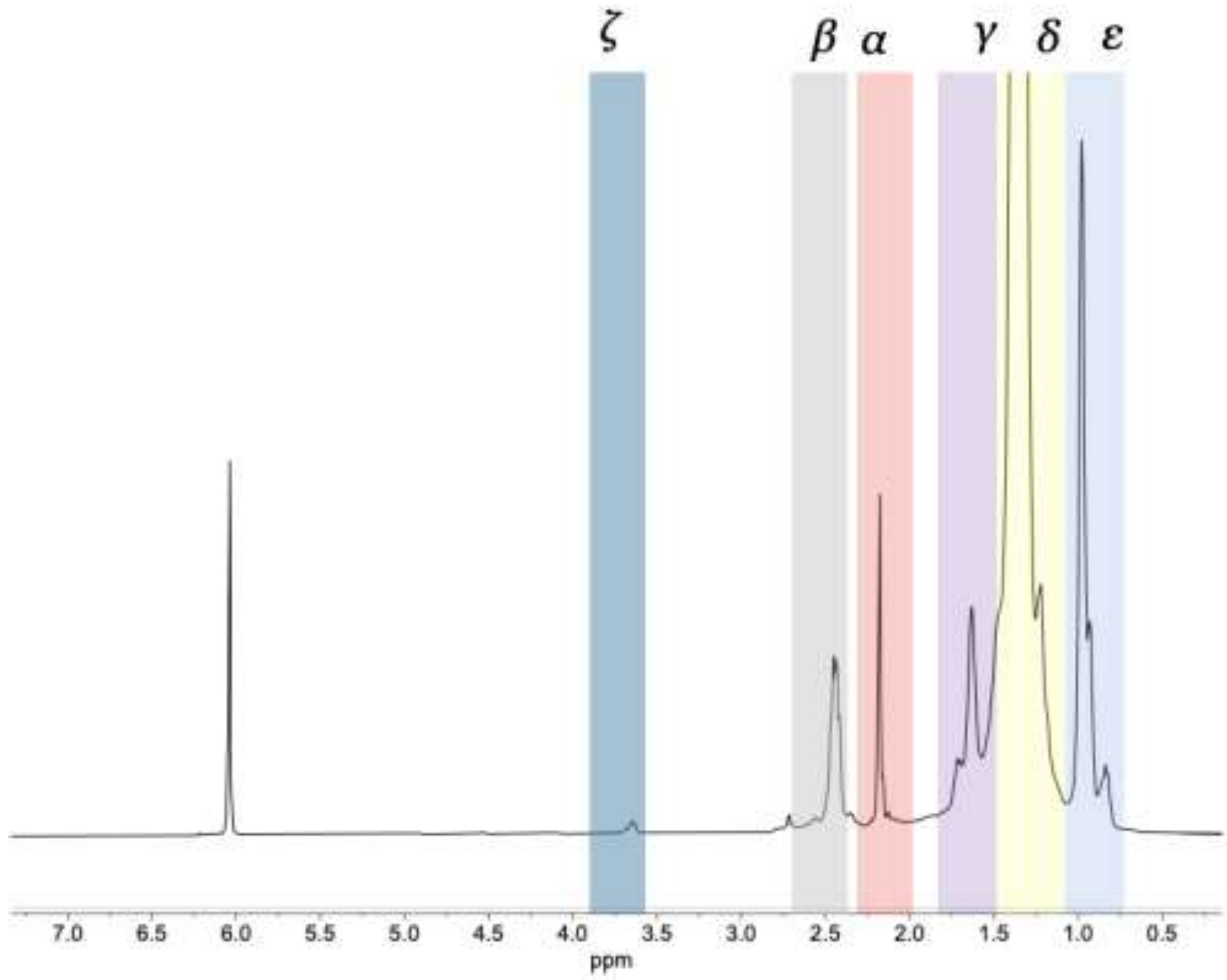

Figure S17. ^{1}H-NMR spectrum of PE4k reaction at 125 °C with HOAc (2.5 mol%) at 0.1 MPa O_2 for 18 hours.

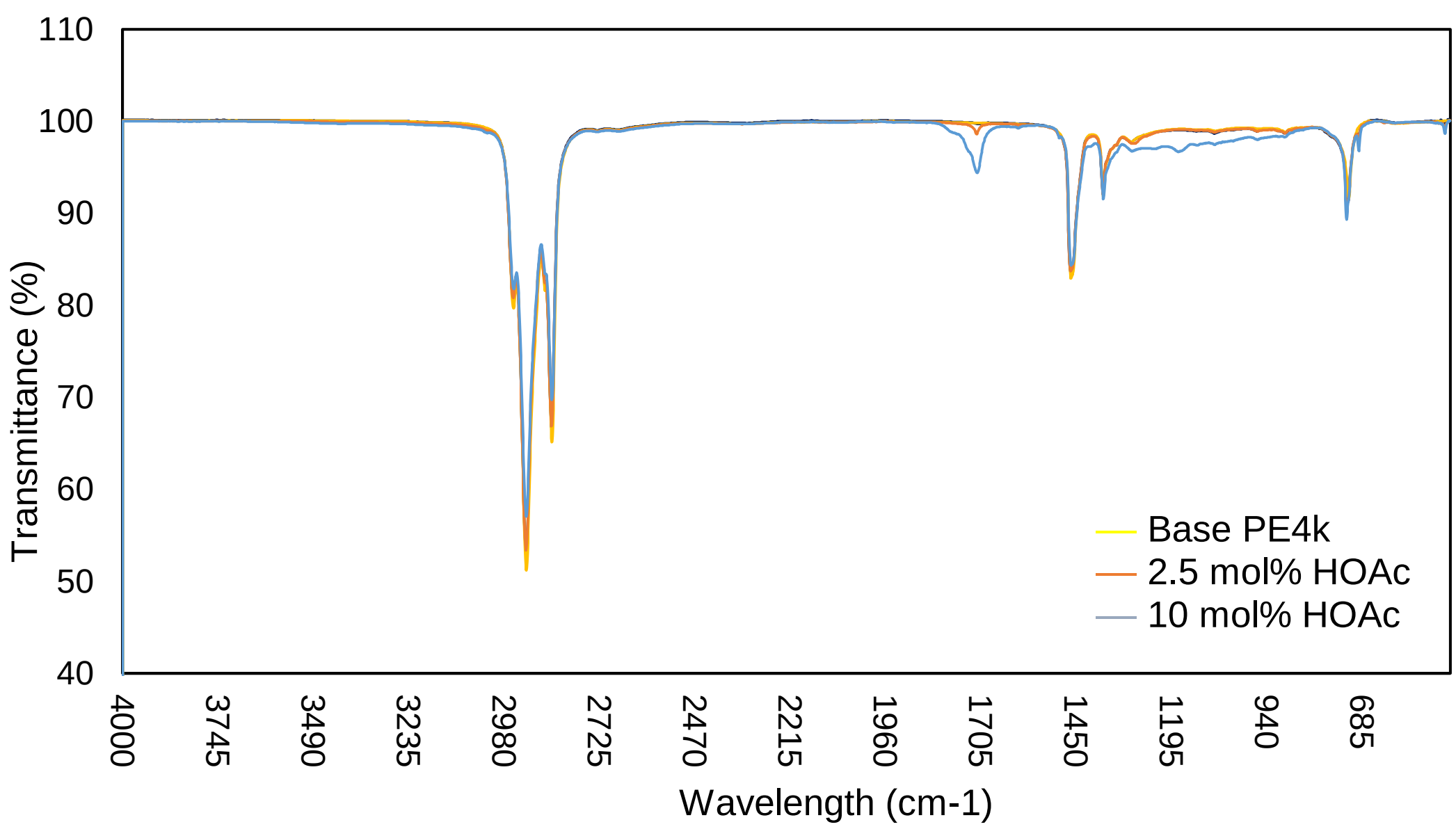

Figure S18. FTIR spectrum of base PE4k and PE4k reaction at 125 °C with HOAc (2.5 mol% and 10 mol%) at 0.1 MPa O_2 for 18 hours. Samples were dried in vacuo before analysis to remove excess HOAc from reaction.

Theoretical calculation of product conversion

Theoretical C_{20} stoichiometric conversion calculation based on the AIBN amount

If we assume the oxidation reaction is a stoichiometric reaction, the converted C_{20} should base on the radical amount formed by 2,2'-Azobis(2-methylpropionitrile) (AIBN). The molecular weight of AIBN is 164.21 g/mol, and the weight of the added AIBN was 50mg.

$$n = \frac{m}{M}$$

There were 3.04×10^{-4}mol AIBN added to the reaction and formed 6.08×10^{-4}mol radicals because each AIBN forms 2 radicals.

The reaction contained 3.0 g *n*-eicosane (M_w = 282.55 g/mol), corresponding to 10.62×10^{-4}mol of substrate. Assuming complete decomposing of AIBN, and one oxidation reaction initiated per radical, the maximum stoichiometric conversion associated with the radical concentration is calculated as:

$$X_{theoretical} = \frac{2n_{AIBN}}{n_{C_{20}}} \times 100\% = \frac{6.08 \times 10^{-4} mol}{10.62 \times 10^{-4} mol} \times 100\% = 5.7\%$$

This calculated conversion based on the strictly stoichiometric initiation model and does not account for radical chain propagation, radical recombination, and termination reactions.

Calculation of CH_2% conversion from C_{20} product conversion

The primary products obtained during oxidation of C_{20} were ketones and (C_{20}-one) and alcohols (C_{20}-ol). The C_{20} conversion was determined from the GC-MS data using the ratio of the total product peak area to that of the unreacted C_{20} plus the products:

$$Conversion = \frac{Peak\ area\ of\ C_{20}one\ +\ C_{20}ol}{Peak\ are\ of\ C_{20}\ +\ Peak\ area\ of\ C_{20}one\ and\ C_{20}ol}$$

The peak area of C_{20}-one and C_{20}-ol obtained from the GC-MS measurements were based on the total ion current (TIC) signal. For structure and molecular weight similar compounds, the TIC response is expected to be similar. Therefore, we assumed similar GC-MS area sensitivity factors for C_{20} and its oxygenated products and calculated the conversion by directly comparing products peak areas with the total peak areas.

The fraction of the CH_2 groups that were converted to ketone and alcohol functional groups and was determined from ^{1}H-NMR spectra as follows:

$$CH_2\ conversion\,\% = \frac{\sum 2'one + \sum internal'one + \sum alcohol + \sum aldehyde}{\frac{1}{2}\sum normal\ CH_2 + \frac{1}{3}\sum normal\ CH_3 + \frac{1}{2}\sum 3'CH_2\ to\ ketone + \frac{1}{2}\sum CH_2\ adjacenet\ to\ carbonyl\ group + \frac{1}{3}\sum end\ CH_3 + \sum aldehyde + \sum alcohol}$$

To demonstrate that our analysis of functional groups and conversion based on ^{1}H-NMR and GC-MS is reasonable, we further correlated the quantitative results from the two methods. Assuming that each C_{20} molecule contains only one site that becomes oxidized to form a hydroxyl or carbonyl group, we can use the C_{20} product conversion obtained from GC-MS to roughly estimate the corresponding CH_2 conversion in each reaction using the following equation:

$$CH_2\ conversion\ in\ NMR\ \% = \frac{C_{20}GCMS\ products\ conversion}{20-C_{20}GCMS\ products\ conversion} \times 100\%$$

For the ^{1}H-NMR spectra, we calculated the proportion of each functional group and its corresponding conversion by integrating each characteristic peak and using the respective peak areas using following equations:

$$\sum 2'one\,\% = \sum end\ CH_3\ \%$$

$$internal'one\,\% = \frac{\sum CH_2\ adjacenet\ to\ carbonyl\ group - \sum end\ CH_3 - \sum aldehyde}{2}$$

The CH_2 group conversion for PE4k was determined using ^{1}H-NMR in a similar fashion.

Mass balance calculation based on the oxygen pressure change in the reactor

Since the oxygen pressure in the batch reactor was measured as a function of time, we can use these data to perform an oxygen mass balance. Assuming that 1 mol of O_2 provides two oxygen atoms and that each oxygen atom is incorporated into one CH_2 unit to form a single oxygen-containing functional group (e.g., an alcohol or ketone), the theoretical CH_2 group conversion can be estimated as:

$$CH_2\ conversion\% = \frac{2n_{O_2,\ comsumed}}{n_{CH_2,\ total}} \times 100\%$$

To estimate the oxygen consumption, the pressure change measured during the reaction was analyzed using the ideal gas law. Taking the experiment shown in Figure 1 as an example, for reaction at 110 °C, the oxygen pressure for the AIBN-assisted reaction (red trace) decreased from 0.69 MPa to approximately 0.37 MPa. Considering the volume occupied by 3 g of eicosane, the gas-phase volume in the 50 mL reactor was estimated to be approximately 47 mL. The amount of oxygen consumed can therefore be calculated from:

$$\Delta nO_2 = \frac{\Delta PV}{RT}$$

where $T = 110 + 273 = 383\ K,\ V = 47\ mL\ =\ 4.7 \times 10^{-5} m^3$, and $\Delta P = 0.32\ MPa = 3.2 \times 10^5\ Pa.$

$$\Delta nO_2 = \frac{(3.2 \times 10^5)(4.7 \times 10^{-5})}{8.314 \times 383} = 4.72 \times 10^{-3} mol$$

Each eicosane molecule contains 18 CH_2 groups. Therefore, 3 g of eicosane (0.0106 mol) contains approximately 0.191 mol of CH_2 units. The corresponding theoretical CH_2 conversion is:

$$CH_2\ conversion\% = \frac{2n_{O_2,\ comsumed}}{n_{CH_2,\ total}} \times 100\% = \frac{4.72 \times 10^{-3} \times 2\ mol}{0.19\ mol} \times 100\% = 4.9\%$$

This theoretical conversion agrees well with the CH_2 conversion determined by quantitative ^{1}H-NMR (4.9%, Table 4, Entry 4). The close agreement between these two independent estimates supports the proposed oxygen mass balance and indicates that the majority of the consumed oxygen was incorporated into oxygen-containing functional products on the C_{20} backbone. Consequently, the formation of fully oxidized products such as CO and CO_2 is expected to be negligible during the reaction.

References:

[1] K. J. Packer, J. M. Pope, R. R. Yeung, M. E. A. Cudby, *Journal of Polymer Science: Polymer Physics Edition* **1984**, *22*, 589–616.

[2] aA. Barlow, L. Wild, R. Ranganath, *Journal of Applied Polymer Science* **1977**, *21*; bR. Lew, P. Cheung, S. T. Balke, T. H. Mourey, *Journal of Applied Polymer Science* **1993**, *47*.
